# A two-step auxin–GA cross-talk regulates organ formation in tomato

**DOI:** 10.1242/dev.205772

**Published:** 2026-07-30

**Authors:** Alon Israeli, Dov Nir, Ido Shwartz, Matan Levy, Marc W. Schmid, Yogev Burko, Idan Efroni, Naomi Ori

**Affiliations:** ^1^ The Institute of Plant Sciences and Genetics in Agriculture, The Robert H. Smith Faculty of Agriculture, Food and Environment, The Hebrew University of Jerusalem, Rehovot 7610001, Israel; ^2^ MWSchmid GmbH, Hauptstrasse 34, 8750 Glarus, Switzerland; ^3^ The Institute of Plant Sciences, Agricultural Research Organization, Volcani Center, Rishon LeZion 7505101, Israel

**Keywords:** Auxin, Gibberellin, Tomato, Organ development, Hormone cross-talk, Compound leaves

## Abstract

Plant organ formation involves an initiation stage followed by growth, together determining organ shape. The plant hormone auxin regulates both initiation and growth. However, it is still not clear how auxin facilitates these two separate successive stages. Here, we show that in tomato compound-leaf development, auxin promotes leaflet initiation and growth via opposite effects on gibberellin (GA) metabolism. Short auxin treatment induced the expression of GA catabolism genes, while a longer increased auxin response induced the expression of GA biosynthesis genes. GA biosynthesis mutants suppressed auxin-induced blade growth. Leaflet number was reduced by increased GA activity, and local reduction of GA activity led to the formation of ectopic leaflets, indicating that leaflet initiation requires reduced GA. Expression of a GA catabolism gene in initiating leaflets was sufficient to induce ectopic leaflets in the adjacent intercalary region, suggesting a non-autonomous function of GA. Collectively, these results point to a context-dependent dual interaction between auxin and GA in organ formation: auxin reduces GA to enable initiation, and induces GA to promote growth.

## Teaser

**Highlighted Article:** Auxin promotes leaflet initiation and growth by successive, opposite effects on gibberellin during tomato leaf development.

## INTRODUCTION

Plants generate and pattern organs in a continuous manner throughout their life, requiring extensive communication between growing cells and tissues. Leaves are lateral organs produced by the shoot meristems (Poethig, 1997; Fleming, 2006; Braybrook and Kuhlemeier, 2010). In compound leaves, such as those of tomato, leaflets are formed from the leaf margin, and their formation has features in common with leaf formation from the shoot apical meristem (SAM) (Brand et al., 2007; Efroni et al., 2010; Floyd and Bowman, 2010). Leaflets form at specific locations at the leaf margin, and in the intercalary regions between initiating leaflets growth is inhibited (Barkoulas et al., 2008; Blein et al., 2008; Koenig et al., 2009; Ben-Gera et al., 2012, 2016; Israeli et al., 2019). Auxin promotes both initiation and growth in many organs, including leaves (Reinhardt et al., 2000) and leaflets (Barkoulas et al., 2008; Ben-Gera et al., 2012; Israeli et al., 2019; Xiong and Jiao, 2019). Growth inhibition between leaflets or between leaf serrations of simple leaves involves specific spatiotemporal activities of growth-inhibiting transcription factors such as CUC and RCO, as well as inhibition of the auxin response (Bilsborough et al., 2011; Bhatia et al., 2023). The auxin response marks leaflet initiation sites, and auxin application to leaf primordia leads to the formation of ectopic leaflets (Koenig et al., 2009). In the intercalary domain, the Aux/IAA protein ENTIRE/SlIAA9 inhibits the auxin response, thus specifying a growth inhibition domain. In the *entire* (*e*) mutant, ectopic auxin response and blade growth occur in the intercalary domain, leading to a continuous blade and an entire leaf shape (Wang et al., 2005; Koenig et al., 2009; Ben-Gera et al., 2012). However, the distinct molecular processes that mediate the auxin response during leaflet initiation and growth are still unknown (Shi and Vernoux, 2018; Heisler and Byrne, 2020; Cavallari et al., 2021; Cucinotta et al., 2021; Roychoudhry and Kepinski, 2021). In addition to auxin, gibberellin (GA) also promotes cell and tissue growth (Stowe and Yamaki, 1957; Sachs et al., 1959). Auxin promotes GA biosynthesis in many developmental processes (Ross et al., 2000; Weiss and Ori, 2007; Ross and Reid, 2010; DeMason and Chetty, 2011; Hu et al., 2018), such as hypocotyl elongation (Chapman et al., 2012; Oh et al., 2014), root elongation (Fu and Harberd, 2003) and fruit growth (Serrani et al., 2008; De Jong et al., 2009; Hu et al., 2018). However, there are also some reports of induction of both GA biosynthesis and degradation genes by auxin (Frigerio et al., 2006; Ross et al., 2011; Kubalová et al., 2025). As the outcome of auxin application during stem elongation was an elevation of GA content (Ross et al., 2000; O'Neill and Ross, 2002), it was concluded that auxin positively regulates GA levels, in agreement with previous findings (Ross and Reid, 2010).

Here, we aimed to understand how auxin promotes organ formation, and whether its effects on organ initiation and growth occur via common or distinct molecular pathways, using leaflet formation in tomato compound leaves. We found that auxin stimulates both GA catabolism and biosynthesis genes in different developmental timings during early leaf development. A short auxin treatment decreased the expression of GA biosynthesis genes and increased the expression of GA catabolism genes, suggesting that leaflet initiation requires reduced GA. In agreement, mutants in GA catabolism genes from the GA2ox family had fewer leaflets, and local expression of a GA catabolism gene was sufficient for leaflet initiation. Conversely, longer auxin treatment induced GA biosynthesis genes, and GA-deficient mutants suppressed auxin-mediated blade growth. These results point to a two-step regulation of GA metabolism by auxin: reduction of GA during initiation and increase during growth.

## RESULTS

### Auxin successively induces the expression of GA degradation and GA biosynthesis genes

**Fig. 1.**
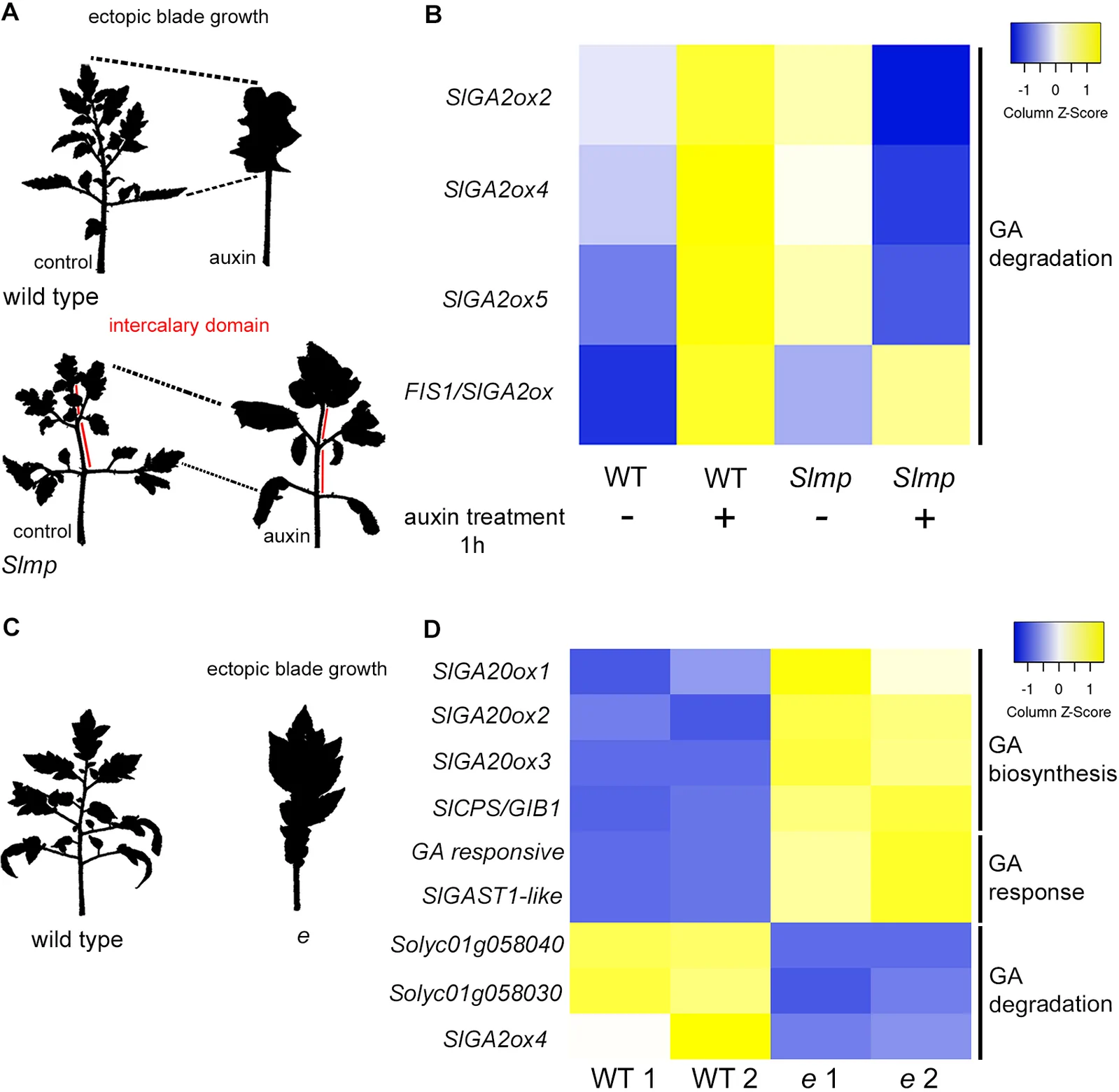
**Auxin promotes blade growth and leaf differentiation.** (A,C) Silhouettes of representative mature leaves of the genotype-treatment combinations used for the RNAseq experiment. While auxin treatment of wild-type leaf primordia results in leaf simplification, *slmp* leaves are relatively resistant to auxin treatment. The dashed lines in the wild type indicate the separated leaflets that turned into one simple blade in response to auxin treatment, and the red lines show the intercalary domain that remained intact in *slmp* leaves after auxin treatment. In C, the ectopic blade growth in fully expanded and developed *e* leaves is shown relative to the wild type. (B) Heat-map showing the relative expression of the *SlGA2ox* family in P5 leaf primordia of wild type and *slmp* with or without 1 h of auxin treatment, assayed by RNAseq. Shown is the average relative expression of the three biological replicates in each genotype-treatment combination. Replicate values were normalized to the size of the libraries. Blue indicates relatively low expression and yellow indicates stronger expression. *Solyc06g068570*, *AP2-like transcription factor*; *Solyc11g066390*, *Superoxide dismutase 3*; *Solyc02g083250*, *Cellular retinaldehyde-binding/triple function*; *Solyc12g045037*, *Omega-6 fatty acid desaturase*. (D) Heat-map showing the increased expression of GA biosynthesis and responsive genes and reduced expression of GA catabolism genes in *e* compared to the wild type, as assayed by RNAseq with two biological replicates. Replicate values were normalized to the size of the libraries. Blue indicates relatively low expression and yellow indicates stronger expression. WT, wild type.

In compound leaves, such as those of tomato, leaflet development proceeds through two temporally distinct phases: an initial stage of localized leaflet initiation at the leaf margin, followed by the expansion and growth of the leaflet blade. While the plant hormone auxin is known to promote both initiation and growth, it remains unclear how a single hormonal signal regulates these two distinct developmental outputs (Koenig et al., 2009; Ben-Gera et al., 2012). To understand how auxin facilitates the successive processes of organ initiation and growth, we profiled the molecular effect of auxin during leaflet formation in tomato compound leaves. To identify the early molecular events induced by auxin in leaflet initiation, we assessed the effect of 1-h auxin application on gene expression in young leaf primordia. The ARF protein SlMP/SlARF5 mediates leaflet initiation and growth downstream of auxin (Fig. 1A; Israeli et al., 2019). Therefore, to focus on genes relevant for auxin-mediated leaflet initiation, we compared auxin-induced gene expression in leaf primordia between wild-type plants and *slmp* mutants. We used P5 leaf primordia of leaf 5, a developmental stage at which leaflets are formed, the expression of *SlMP* is the highest, and leaf primordia are relatively similar between the two genotypes (Fig. S1A; Israeli et al., 2019). Compared to the wild type, in which 378 genes had elevated expression in response to auxin (Table S1A; Lieberman-lazarovich et al., 2019), in *slmp* primordia 158 were elevated. Likewise, 217 were downregulated in the wild type compared to 128 downregulated genes in the *slmp* mutant. In total, 233 genes responded significantly differently to auxin treatment in the wild type compared to the mutant (Table S1A), confirming the prominent role of SlMP in mediating auxin-responsive gene expression in leaflet initiation. To dissect the molecular basis of a long-term increase in auxin response, which promotes ectopic blade growth, we used the *entire* (*e*) mutant, in which there is a loss-of-function mutation in the gene encoding the auxin-response inhibitor ENTIRE/SlIAA9 (E). *e* mutants have ectopic, expanded auxin response and blade growth in the intercalary domain, leading to partial leaflet fusion (Fig. 1C; Wang et al., 2005; Koenig et al., 2009; Ben-Gera et al., 2012). In *e slmp* mutants, the ectopic blade growth phenotype of *e* is suppressed, indicating that this ectopic growth is mediated by SlMP (Israeli et al., 2019). *e* leaf primordia therefore experience prolonged increased auxin response. As the main leaf phenotype in *e* is an expanded, ectopic blade, we reasoned that it may enable the elucidation of the molecular response to auxin that is relevant to blade expansion. We compared gene expression between wild-type and *e* shoot apices containing the meristem and the four youngest leaf primordia (m+P4), when leaf primordia are similar between these genotypes (Fig. S1B). This comparison identified 3090 and 2304 genes with increased or decreased expression in *e*, respectively (Table S2A).

In both comparisons, the expression of multiple GA metabolism genes was affected, but in an opposite manner (Tables S1A,B and S2A,B). A short auxin treatment induced several GA catabolism genes, while in the *e* mutant we observed elevated expression of several GA biosynthesis genes. Following a short auxin treatment, the expression of several genes from the class I *GA2ox* gene family, encoding GA catabolism enzymes (Thomas et al., 1999; Rieu et al., 2008; Chen et al., 2016), was elevated in the wild type but not in *slmp* in response to auxin (Fig. 1B). Class 1 SlGA2ox genes are thus strong candidates as early auxin-responsive genes in leaflet initiation. Induction of several class I SlGA2ox genes by short auxin treatment was confirmed in several experiments, showing their induction as early as 10 min of auxin treatment (Table S3A-D).

In contrast to the induction of GA catabolism genes following short auxin treatment, the expression of many GA biosynthesis genes was elevated in the *e* mutant (Fig. 1D), including *SlCPS/GIB1*, *SlGA20ox1*, *SlGA20ox2* and *SlGA20ox3*, suggesting that a steady-state elevated auxin response leads to increased GA activity. Several GA-responsive genes were also increased in the *e* mutants, while several genes encoding for GA catabolism enzymes were downregulated (Fig. 1D, Fig. S2, Table S2). Together, these transcriptomic profiles suggest that auxin has two distinct, temporally separated, roles during leaflet development: Rapid auxin signaling acts locally at the leaf margin to transiently suppress GA activity and allow leaflet initiation, whereas prolonged auxin signaling promotes GA biosynthesis, which may drive lamina development and broad leaf expansion. In agreement with a role in initiation, the expression of *SlGA2ox4* was previously shown to coincide with that of the auxin response at leaf and leaflet initiation sites (Yanai et al., 2011).

**Fig. 2.**
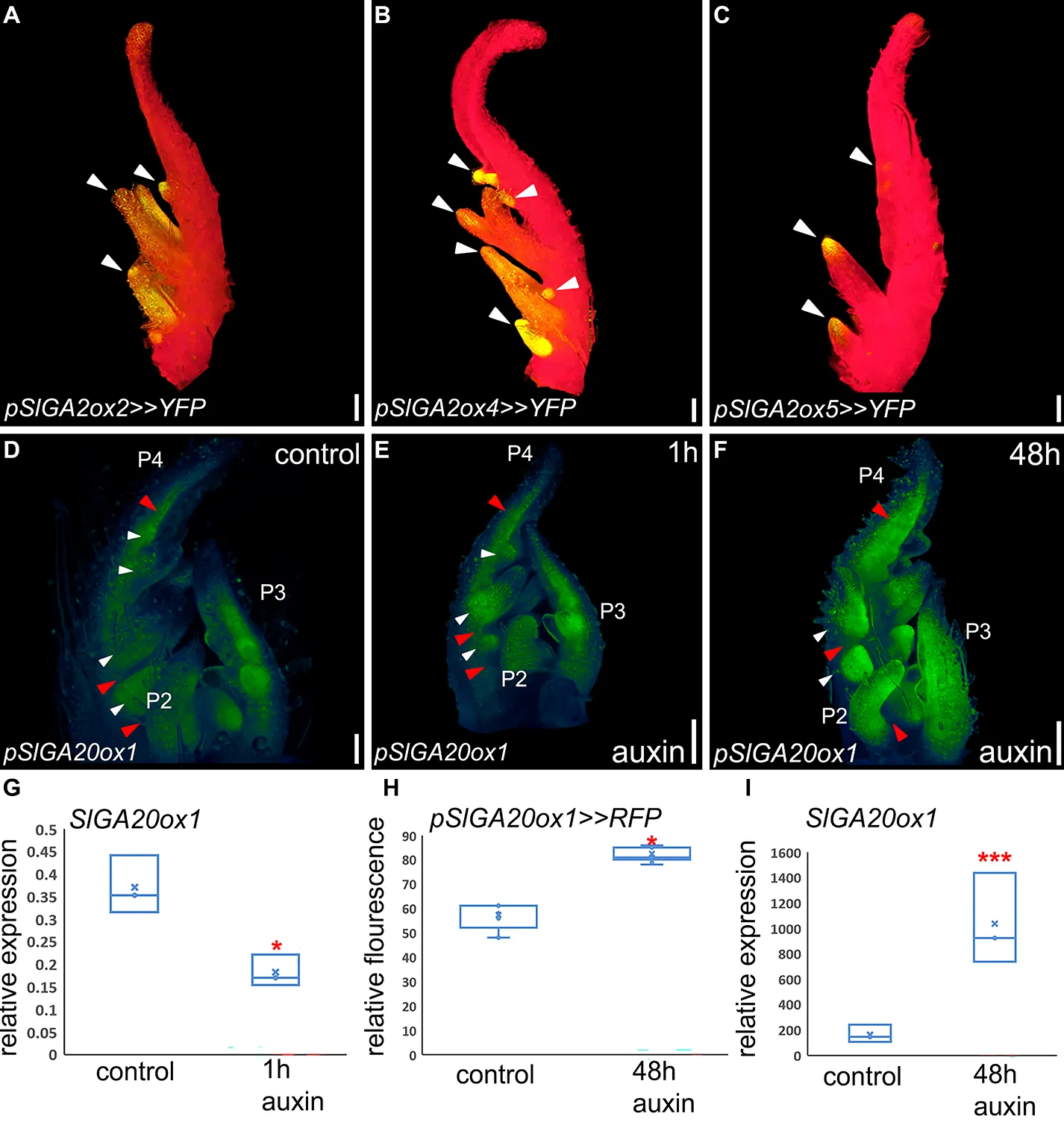
***SlGA2ox* promoters are expressed at leaflet initiation sites and p*SlGA20ox1* in growing areas.** (A-C) Stereomicroscope images showing *pSlGA2ox2*, *pSlGA2ox4* and *pSlGA2ox5* driving a YFP reporter, in wild-type young leaf primordia. White arrowheads point to primary or intercalary leaflets, which coincide with the expression of the *SlGA2ox* promoters in young leaf primordia. (D-F) Stereomicroscope images showing *pSlGA20ox1≫RFP* expression in the wild-type SAM and young leaf primordia with (1 h, 48 h) and without (control) auxin treatment, as indicated. White arrowheads point to primary leaflets. Red arrowheads point to the intercalary domain and to regions of increased *pSlGA20ox1* expression and ectopic blade growth in the distal domain. (G,I) qRT-PCR analysis of *SlGA20ox1* mRNA expression in wild-type shoot apices (m+P5) 1 h and 48 h following auxin application*,* relative to the *EXP* reference gene. (H) Quantification of the relative fluorescence of the *pSlGA20ox1≫RFP* (in D,E) using ImageJ software. In all boxplots, the central horizontal line marks the median, the cross indicates the mean, upper and lower box lines represent the first and third quartile, respectively, and the whiskers represent the maximum and minimum values of at least three biological replicates. *P<0.05, ***P<0.001 (unpaired, two-tailed Student's *t*-test). Scale bars: 200 μm (A-F).

In agreement, *SlGA2ox2*, *SlGA2ox4* and *SlGA2ox5* promoters drove a relatively broad expression in P1-P3 leaf primordia, followed by a more confined expression at leaflet initiation sites and leaflet tips. Notably, two of them were also expressed in initiating lateral roots (Fig. 2A-C, Figs S3 and S4). The promoter of the GA biosynthesis gene *SlGA20ox1* drove expression mainly in growing regions of the leaf, where blade expansion takes place (Fig. 2D, Fig. S5). In addition to the induction of *SlGA2ox*, a short auxin treatment also led to reduced expression of *SlGA20ox1* (Fig. 2D,E,G), further supporting reduced GA levels as an early auxin response in leaflet initiation. Following the initial reduction in *SlGA20ox1* expression 1 h after auxin treatment, its expression was elevated 48 h after auxin treatment, also evidenced by a substantially increased expression of its promoter (Fig. 2D-F,H,I), in agreement with a role for increased GA in tissue growth and expansion. Early tomato leaves progress through development faster than later leaves (Shleizer-Burko et al., 2011). We assessed *GA20ox1* expression at the P5 stage in successive leaves formed on the plants. *SlGA20ox1* expression was higher in L1 and L2 at this stage relative to leaves 4-6, consistent with the faster differentiation of the earlier leaves and with the involvement of SlG20ox1 in blade expansion (Fig. S5C). Overall, these results imply a dual interaction between auxin and GA in leaflet development. Leaflet initiation involves reduced GA activity, achieved by auxin-induced elevation of *SlGA2ox* expression and reduction of *SlGA20ox*1 expression via SlMP, while auxin-mediated blade growth involves increased GA activity, resulting from an increased expression of GA biosynthesis genes.

### GA catabolism and biosynthesis genes promote auxin-mediated leaflet initiation and expansion, respectively

**Fig. 3.**
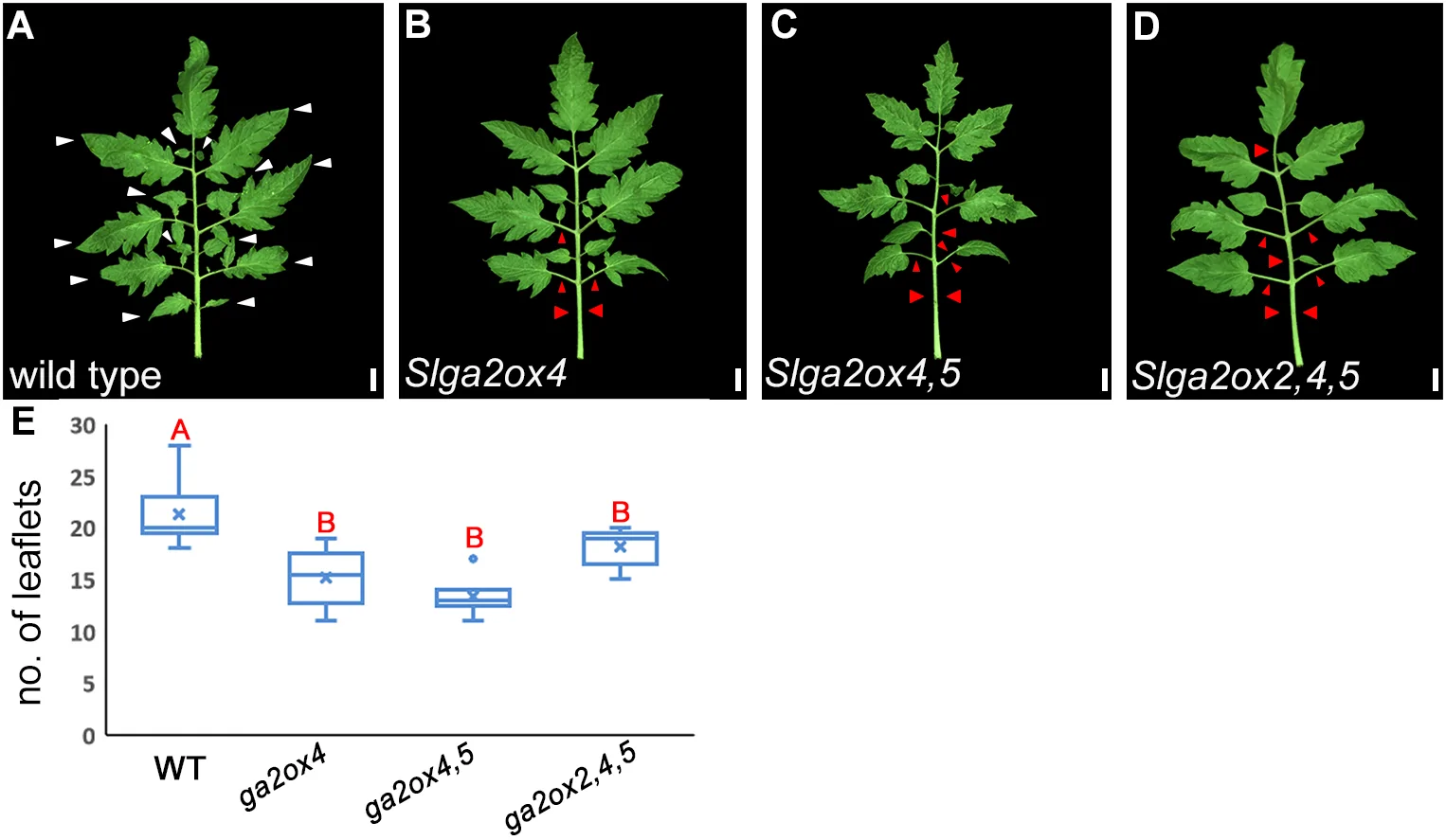
**Mutations in SlGA2ox genes reduce leaflet number.** (A-D) Mature fifth leaves of the indicated genotypes. White arrowheads point to primary and intercalary leaflets. Red arrowheads point to missing leaflets. Scale bars: 2 cm. (E) Quantification of the total number of leaflets in the indicated genotypes in A-D. Boxplots of at least five leaves (see Fig. 1 legend for definition of boxplot components). Different letters indicate statistically significant differences determined by one-way ANOVA followed by Tukey–Kramer's multiple comparisons test (*P*<0.05). WT, wild type.

To genetically test the role of SlGA2ox in leaflet initiation, we generated mutant combinations in three closely related class 1 SlGA2ox genes that were highly induced by 1 h auxin treatment in wild-type but not *Slmp* plants, *SlGA2ox*2, *SlGA2ox4* and *SlGA2ox5*, and in two more distant family members, *SlGA2ox8* and *SlGA2ox11* using CRISPR/CAS9-mediated gene editing (Fig. S6). We also characterized leaf development in an available class 3 *Slga2ox7* mutant (Schrager-Lavelle et al., 2019). *Slga2ox7*, *Slga2ox8* and *Slga2ox11* did not show any aberrant leaf phenotype (Fig. S7). In contrast, the class 1 *Slga2ox4* mutant had a reduced number of leaflets compared to the wild type. Double *Slga2ox4,5* and triple *Slga2ox2,4,5* mutants also had fewer leaflets than the wild type, but there was no further reduction compared to the single *Slga2ox4* mutant (Fig. 3). Elevated GA activity was previously shown to lead to reduced leaflet number, likely by accelerating leaf maturation and shortening the morphogenetic window during which leaflet initiation occurs (Jasinski et al., 2008; Yanai et al., 2011; Israeli et al., 2021). The induction of GA catabolism genes and reduction of GA biosynthesis genes by a short auxin treatment of young, morphogenetic leaf primordia suggested that reduced GA is also required locally within the morphogenetic window, for leaflet initiation downstream of auxin signaling. To address this possibility, we examined the effect of GA treatment in the *slmp* mutant background. GA treatment further reduced leaflet number in *slmp* leaves (Fig. S8A-E,L). Furthermore, *slmp* mutants showed an additional reduction in leaflet number in response to GA application at relatively low concentrations compared to the wild type (Fig. S8). The additional reduction may result from GA being regulated by other factors in addition to auxin and SlMP. While it is challenging to distinguish between the global and local effects of GA, these findings support the requirement for reduced GA in auxin-mediated leaflet initiation. Overall, this suggests that GA affects leaflet number both by shortening the morphogenetic window during which leaflets are formed and by locally inhibiting leaflet initiation within this developmental context.

**Fig. 4.**
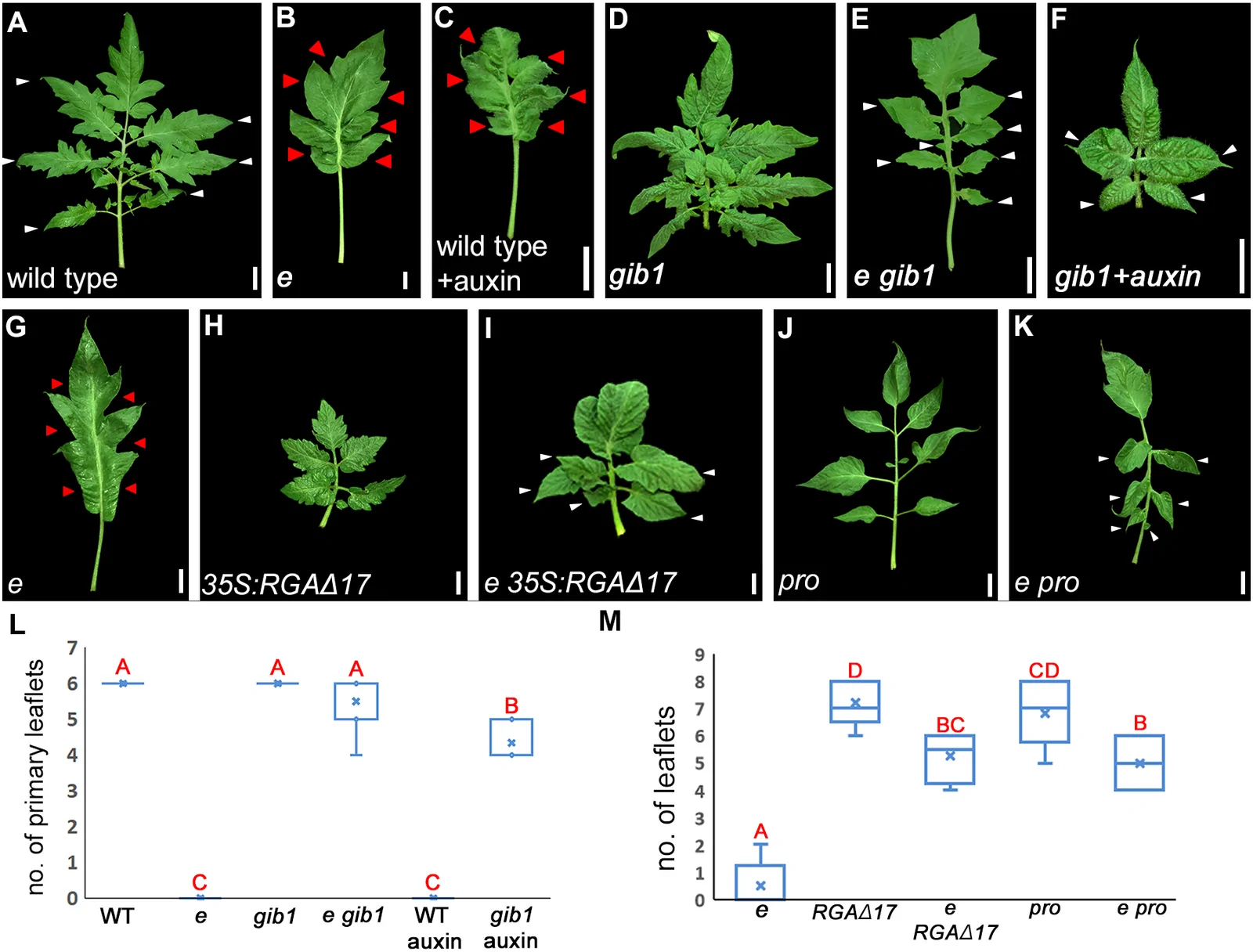
**GA mediates auxin-induced ectopic blade growth.** (A-K) Mature fifth leaves (A-F) and later leaves (produced after the transition to flowering) (G-K) of the indicated genotypes. Scale bars: 2 cm. White arrowheads point to primary or intercalary leaflets and red arrowheads to fused ectopic blade in the intercalary domain. (L,M) Quantification of the number of leaflets in the indicated genotypes and treatments. Boxplots of at least five leaves (see Fig. 1 legend for definition of boxplot components). Different letters indicate statistically significant differences determined by one-way ANOVA followed by Tukey–Kramer's multiple comparisons test (*P*<0.05). WT, wild type.

The upregulation of GA biosynthesis genes in *e* and following prolonged auxin application suggested that auxin promotes blade growth by elevating GA levels. In agreement with increased GA activity in *e* leaves, they showed characteristics of enhanced GA response, such as a paler color, reduced chlorophyll level and fewer trichomes (Fig. S9). If GA is required for auxin-mediated blade growth, then reduced GA is expected to suppress the ectopic blade growth seen in *e* mutants or in response to auxin application. To genetically test this prediction, we used mutants impaired in GA biosynthesis. *gib1/slcps* (Hu et al., 2018), *gib2* and *gib3* are classical tomato mutants, which do not produce GA due to mutations in early GA biosynthesis genes (Fig. S2; Koornneef et al., 1990; Hu et al., 2018). As shown before, *gib* mutants did not affect total leaflet number but produced more secondary leaflets per primary leaflet (Fig. 4D,L, Fig. S10; Jasinski et al., 2008). However, *gib* mutants suppressed the ectopic blade growth of *e* mutants or following auxin treatment, and this effect was eliminated by GA application (Fig. 4A-F,L, Fig. S10). This indicates that ectopic blade growth in response to increased auxin activity depends on GA. Collectively, these results provide genetic support for the importance of GA biosynthesis in promoting auxin-mediated blade growth within the morphogenetic window.

The results suggest that auxin promotes leaflet formation by reducing and elevating GA response at initiation and expansion, respectively. In agreement with this dual effect of auxin on GA, both *della/pro* loss of function and overexpression of the stable DELLA/RGA from *Arabidopsis* suppressed the ectopic blade growth of *e* (Fig. 4G-K,M).

### Local and transient reduction of GA response facilitates leaflet initiation

**Fig. 5.**
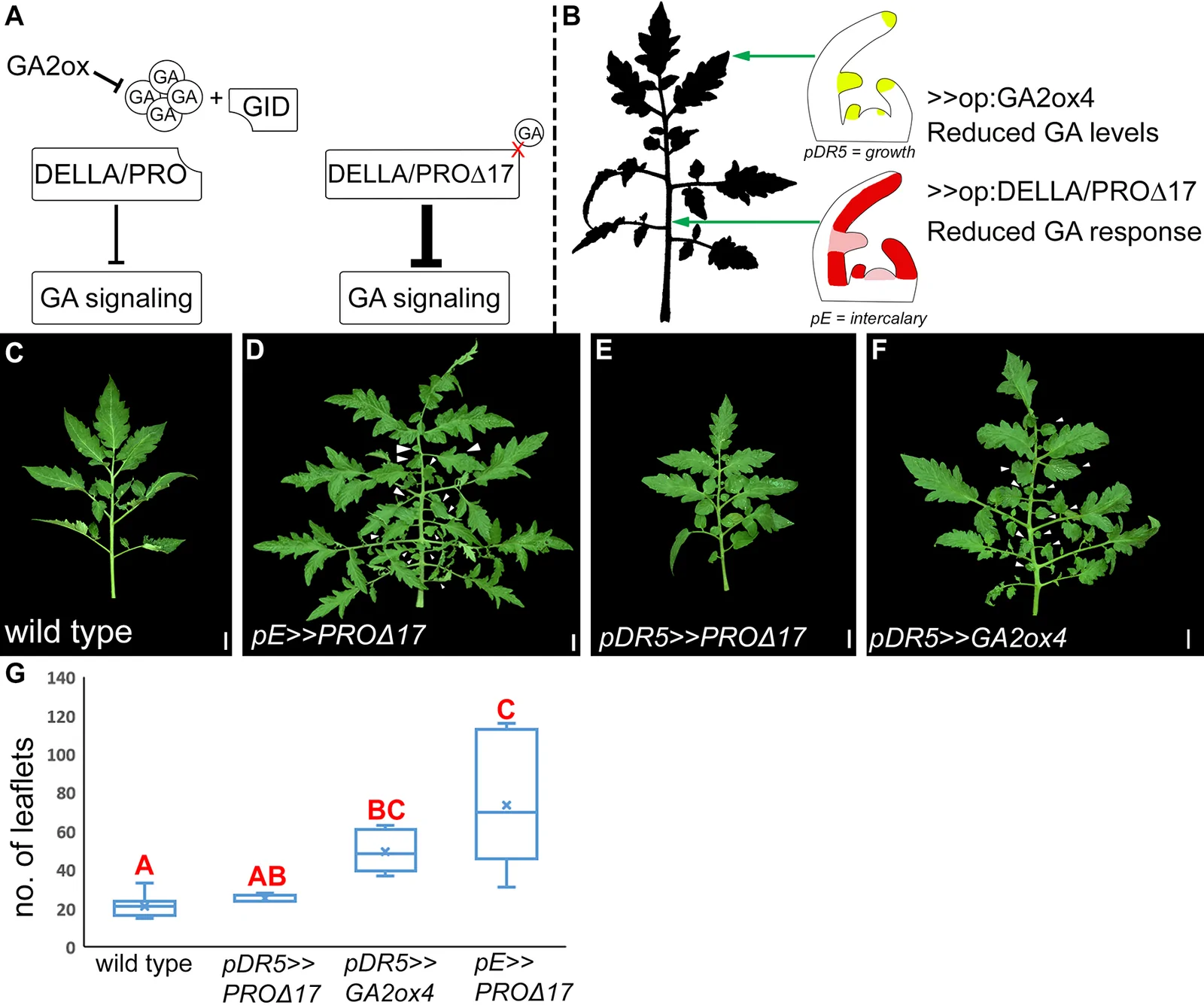
**Reduced GA response is sufficient to induce leaflet formation.** (A) Schematic of the roles of GA2ox and PRO in GA metabolism and response. The tomato DELLA protein PROCERA (PRO) inhibits GA response, and GA promotes its degradation. *SlGA2ox4* encodes a GA degradation enzyme that inactivates GA, thus reducing GA levels. PROΔ17 is a mutated PRO version that constitutively inhibits GA response. (B) Schemes of the expression domains of the *pE* and *pDR5* promoters and the corresponding domains in a representative fully expanded wild-type leaf. Stronger color represents stronger expression. The *E* promoter drives expression mainly in the intercalary domain between primary leaflets. The *DR5* promoter drives expression in leaflet initiation sites. (C-F) Mature fifth leaves of the indicated genotypes. White arrowheads point to ectopic and normal primary or intercalary leaflets. Scale bars: 2 cm. (G) Quantification of the total number of leaflets in the indicated genotypes in C-F. Boxplots of at least three leaves (see Fig. 1 legend for definition of boxplot components). Different letters indicate statistically significant differences determined by one-way ANOVA followed by Tukey–Kramer's multiple comparisons test (*P*<0.05).

The findings presented above suggested that leaflet initiation requires a transient local reduction in GA response, which is realized by a rapid elevation of GA2ox activity and reduced GA20ox1 activity at the sites of leaflet initiation in response to auxin. We therefore aimed to reduce GA in the specific domains of differential growth at the leaf margin, and test whether local reduction of GA activity would induce leaflet initiation. To alter GA level or response in the intercalary domain, where normally there is no leaflet formation, we used the *E* promoter, which drives expression mainly in the intercalary domain (Fig. 5A,B; Israeli et al., 2019). For the initiation domain, we used the *DR5* promoter, expressed locally at the sites of leaflet initiation. *pE*≫*PROΔ17* plants, expressing a GA-insensitive form of the GA-response inhibitor PRO, produced leaves with many ectopic leaflets in the intercalary domain (Fig. 5C,D,G). The leaves were longer with more and longer primary leaflets. In contrast, *pDR5*≫*PROΔ17* did not affect leaf shape, as could be expected from the predicted endogenous reduction of GA response in this domain at leaflet initiation (Fig. 5E,G). Therefore, local reduction of GA response in the intercalary domain but not the initiation domain is sufficient to induce ectopic leaflet initiation. To test whether GA acts autonomously, we expressed the GA catabolism gene *GA2ox4* in the initiation domain. In contrast to *pDR5*≫*PROΔ17*, *pDR5*≫*GA2ox4* leaves produced many ectopic leaflets in the intercalary domain (Fig. 5F,G). Therefore, reduction of GA response produces ectopic leaflets locally, while reduction of GA level acts non-autonomously. Together, these results suggest that GA acts in a partially non-autonomous manner to inhibit leaflet initiation in the intercalary domain, and reduction in GA levels in either the initiation or the intercalary domain leads to ectopic leaflet initiation in the intercalary domain.

## DISCUSSION

**Fig. 6.**
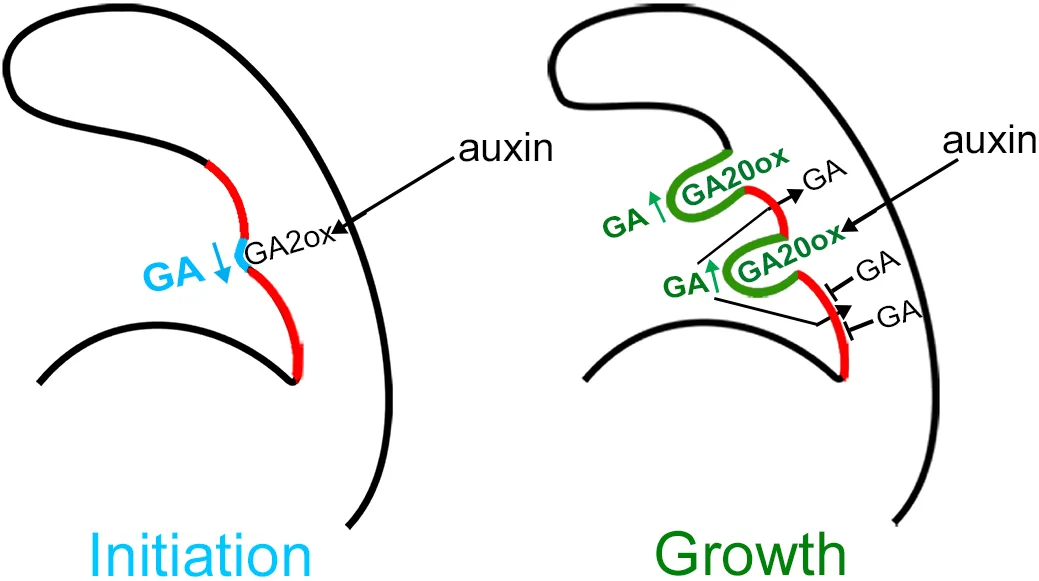
**Model for auxin–GA interaction during compound-leaf development.** Leaflet formation proceeds through two successive stages regulated by auxin via opposite effects on GA metabolism. In young leaf primordia (stage 1), high auxin response at the initiation sites (light blue) induces the expression of GA catabolism genes of the *GA2ox* family, leading to a local reduction in GA levels and/or response. This transient decrease in GA activity permits leaflet initiation. In the adjacent intercalary region (red), GA activity remains high and inhibits leaflet initiation. During the subsequent growth phase (stage 2), sustained auxin signaling promotes the expression of GA biosynthesis genes, including GA20ox genes, resulting in elevated GA levels that drive blade growth in the growth domain (green). GA may act non-autonomously, moving from growing leaflets to the intercalary domain to maintain inhibition of leaflet initiation. Thus, auxin first enables initiation by reducing GA and later promotes growth by increasing GA, while GA acts locally and non-autonomously to restrict leaflet initiation in the intercalary region.

Using compound leaf development as a model, we uncovered a unique, two-step regulation of GA by auxin in organ formation. We suggest that auxin facilitates leaflet formation in two steps: first, a rapid response in which auxin promotes leaflet initiation by reducing the activity of GA-biosynthesis genes and increasing the activity of GA-catabolism genes, and second, a slower, steady-state response in which auxin promotes blade growth and expansion by increasing GA biosynthesis (Fig. 6).

### Successive opposite interactions between auxin and GA in leaflet initiation and growth

The dual, two-step interaction between auxin and GA shown here is intriguing due to the mostly positive interaction between these two hormones (Depuydt and Hardtke, 2011; Ross et al., 2011; Mazzoni-Putman et al., 2021), the many examples of a promoting effect of auxin on GA biosynthesis (Ross et al., 2000; Wolbang and Ross, 2001; O'Neill and Ross, 2002; Serrani et al., 2008; De Jong et al., 2011; Hu et al., 2018), and the opposite direction of the interaction in the two phases. However, these two steps in organ development involve distinct developmental bases at the cellular level, and therefore distinct regulation may be required. Organ initiation, which mainly involves cell-wall changes and cell division (Hagemann and Gleissberg, 1996; Fleming, 2006), may require reduced GA, in agreement with the requirement for reduced GA activity in SAM maintenance (Jasinski et al., 2005; Fleming, 2006). Conversely, during blade growth there is extensive cell expansion (Donnelly et al., 1999; Dengler and Tsukaya, 2001), which is promoted by GA (Stowe and Yamaki, 1957; Feucht and Watson, 1958; Achard et al., 2009). The interaction between auxin and GA identified here is therefore adjusted to the specific events occurring at these two distinct stages of leaflet development.

As auxin has been shown to induce mostly the expression of GA biosynthesis genes, such as *GA20ox* (Desgagné-Penix and Sponsel, 2008; Serrani et al., 2008; Hu et al., 2018), it is generally accepted that auxin promotes GA biosynthesis. However, several reports noted that auxin occasionally also induces the expression of GA2ox genes (O'Neill and Ross, 2002; Frigerio et al., 2006; Serrani et al., 2008; Liu et al., 2018; Kim et al., 2020; Kubalová et al., 2025). These studies mostly concluded that the main effect of auxin is to increase GA levels, and therefore interpreted the *GA2ox* induction by auxin as a feedback effect of the elevated GA or a result of the common origin of GA20ox and GA2ox (Ross and Reid, 2010). However, Frigerio et al. (2006) showed a relatively fast induction of some GA2ox genes by auxin in *Arabidopsis* seedlings. They proposed that auxin may induce *GA20ox* and GA2ox genes in different tissues or induces them together in the same tissue to enable fine-tuning of GA activity (Frigerio et al., 2006). In agreement, here GA2ox genes were induced as early as 10 min after auxin treatment. Recently, auxin was shown to control *Arabidopsis* root elongation by reducing GA levels through induction of At*GA2ox6* and At*GA2ox8* (Kubalová et al., 2025). In contrast to *Arabidopsis* seedlings, here we find a temporal gap between the early induction of GA2ox genes, accompanied by reduced expression of *GA20ox*, and the later induction of *GA20ox*, which enables initiation and expansion, respectively.

GA2ox genes were shown to be expressed punctually at initiation sites of other organs, such as leaves, flowers and lateral roots (Yanai et al., 2011; Li et al., 2019; Kinoshita et al., 2020), the initiation of which is promoted by auxin (Reinhardt et al., 2000; Fukaki et al., 2002; Yamaguchi et al., 2013; Capua and Eshed, 2017). In agreement with a role in organ initiation, overexpression of GA2ox genes or a DELLA GA-response inhibitor in *Populus tremula* (aspen) led to an increased number of lateral roots, while GA application reduced the number of lateral roots and suppressed the effect of *GA2ox* and DELLA overexpression (Gou et al., 2010). These results support a functional role for the reduction in GA activity during lateral root initiation, as shown here for leaflet initiation. Additionally, reduced GA activity was also shown to mediate the promoting effect of auxin on ovule initiation (Gomez et al., 2018), which occurs within the ovary at sites equivalent to leaf margins (DeMason and Chetty, 2011; Van Valen, 1982; Cucinotta et al., 2014; Hawkins and Liu, 2014; Hepworth and Pautot, 2015). This supports a more general role for GA2ox in organ initiation and suggests that the two-step action of auxin via GA shown here for leaflet patterning may also hold for other organs. A two-phase opposing role was demonstrated for GA in the transition to flowering (Yamaguchi et al., 2014). In this study, GA was shown to promote the transition from vegetative to reproductive development, which involves stem elongation, but to inhibit the further formation of flowers, involving organ initiation.

### GA regulates organ development in multiple pathways

The duration of the morphogenesis window in compound tomato leaves, which has a central role in leaf patterning, has been reported to depend on the balance between cytokinin (CK), which promotes morphogenesis, and GA, which promotes differentiation (Shani et al., 2010; Fleishon et al., 2011; Yanai et al., 2011; Bar and Ori, 2014; Shwartz et al., 2016; Israeli et al., 2021). This role is reflected by the relatively broad expression of the GA biosynthesis gene *SlGA20ox1* (Figs 2 and 4). Patterning events that determine the shape of the leaf margin by differential growth, including the formation of leaflets, occur within this morphogenetic window (Bar and Ori, 2014; Israeli et al., 2020). Global GA level or response was previously shown to affect the dynamics of leaf differentiation by altering the duration of the morphogenetic window and the dynamics of cell growth, consequently affecting overall organ size and form. For example, *pro* mutant primordia elongate earlier than the wild type by cell enlargement. While the GA biosynthesis mutant *gib1* did not affect total leaflet number, it had an increased ratio of secondary to primary leaflets, and enhanced KNOX gain-of-function phenotypes, suggesting that KNOX and GA act in partially parallel pathways to regulate the morphogenetic window (Jasinski et al., 2008; Yanai et al., 2011; Israeli et al., 2021). The current results indicate that, in addition to its effect on the overall differentiation rate and the morphogenetic window, GA is also involved in local patterning downstream of auxin. Using local genetic perturbations to reduce GA response or levels enabled the separation of these two roles, revealing that a local reduction of GA activity is sufficient to induce ectopic leaflet initiation (Fig. 5). Furthermore, the suppression of ectopic blade growth in *e* mutants or following auxin treatment by *gib* mutants, provides genetic evidence that auxin-mediated blade expansion requires GA biosynthesis (Fig. 4). These findings suggest that spatial juxtaposition of low- and high-GA domains contributes to leaflet patterning similarly to local auxin distribution patterns (Barkoulas et al., 2008; Koenig et al., 2009; Bilsborough et al., 2011; Ben-Gera et al., 2012), and that GA thus has important roles in both global differentiation timing and local organogenesis. The broad effects of global GA deficiency on cell expansion and leaf maturation likely masked the more local role of GA in leaflet initiation within the morphogenetic window. Thus, GA has important roles in both tuning global differentiation and local patterning. CK was recently shown to locally repress growth in leaflet bases downstream of the REDUCED COMPLEXITY (RCO) homeodomain protein (Hajheidari et al., 2019). Furthermore, auxin was shown to promote global leaf differentiation in *Cardamine hirsuta* in addition to its known role in leaf marginal patterning (Kierzkowski et al., 2019). Therefore, it appears that auxin, GA and CK each affect both global differentiation and local patterning.

## MATERIALS AND METHODS

### Plant material and growth conditions

Tomato [*Solanum lycopersicum* cv. M82 (LA3475)] plants were used throughout the study. The following genotypes were described previously: *slmp* and *pE:LhG4* #2 (Israeli et al., 2019); *DR5:VENUS* (Shani et al., 2010); *op:RFP* (Shalit et al., 2009); *gib1*, *gib2* and *gib3* (Bensen and Zeevaart, 1990; Koornneef et al., 1990); *op:GA2ox4* (Yanai et al., 2011); *op:PRO*Δ*17* #1 (Nir et al., 2017); *slga2ox7* (Schrager-Lavelle et al., 2019); *35S:RGA*Δ*17* (Livne et al., 2015); *pro* (Yanai et al., 2011); *e-4* (Berger et al., 2009). The promoters (*pSlGA20ox1*, *pSlGA2ox2*, *pSlGA2ox4* and *pSlGA2ox5*) and the mutants (*slga2ox2*, *slga2ox4*, *slga2ox5*, *slga2ox8* and *slga2ox11*) were generated during this study and are described below. Seeds were germinated and grown in a growth room or a growth chamber for 2-4 weeks. The seedlings were then transferred to a greenhouse with natural daylight and temperature.

### Cloning and trans-activation system

For transgenic expression of genes and sensors under the control of specific promoters, we used the Promoter:LhG4 (p) and Operator (OP) transactivation system as described before (Moore et al., 1998; Shani et al., 2009). Briefly, in this system, driver lines expressing the synthetic transcription factor LhG4 under the control of a specific promoter are crossed to responder lines containing a gene of interest under the control of the *Escherichia coli* operator, which is recognized by the LhG4 transcription factor but not by any endogenous plant transcription factor. The resulting F1 plants express the selected gene under the control of the selected promoter (*p*≫*GENE*). For driver lines, fragments upstream of the transcription start sites (TSS/ATG), designated ‘p’, were amplified from M82 genomic DNA as follows: *pSlGA20ox1*, 2633 bp; *pSlGA2ox2*, 1739 bp; *pSlGA2ox4*, 1541 bp; *pSlGA2ox5*, 3076 bp, which included 78 bp downstream of the ATG. The fragments were cloned into the pENTR-D-TOPO (Invitrogen) entry vector, followed by LR recombination into the LhG4 binary vector. For the *pDR5:LhG4* driver line, the synthetic *pDR5* promoter was digested out of the direct fusion construct *pDR5:VENUS* (Shani et al., 2010; Ben-Gera et al., 2012) using BamHI and KpnI restriction sites. The resulting fragment was inserted into a plasmid containing the *LhG4* sequence to generate the *pDR5:LhG4* binary vector. For the *op:PRO*Δ*17* responder line, we used a previously published line described by Nir et al. (2017). Two independent lines were characterized with different promoters and showed similar phenotypes with varying severity. To avoid variability resulting from differences in transgene strength, one representative line was selected and used consistently throughout all crosses and experiments. For the *pE:LhG4* driver line, we used a previously published line described by Israeli et al. (2019). Two independent lines were tested in a cross with *op:PRO*Δ*17* and showed similar phenotypes with varying severity. To avoid variability resulting from differences in transgene strength, one representative line was selected and used consistently throughout all crosses and experiments. For the *op:GA2ox4* responder line, we used a previously published line described by Yanai et al. (2011), which was used consistently throughout the study. For the *pDR5:LhG4* driver line, we used a line showing expression patterns similar to the direct fluorescent *DR5:VENUS* reporter described previously (Shani et al., 2010; Ben-Gera et al., 2012). Following the generation of transgenic plants, at least two independent lines from each construct were crossed to an op:YFP or op:RFP responder line. A representative line was selected for further analysis following characterization of the expression domains. The *pE*≫*PRO*Δ*17*, *pDR5*≫*PRO*Δ*17*, *pE*≫*GA2ox4* and *pDR5*≫*GA2ox4* lines were generated using the trans-activation system from previously described driver and responder lines (see ‘Plant material and growth conditions’ section). Primers used for cloning, qRT-PCR and CRISPR-mediated editing are listed in Table S4.

### Hormone treatments

For auxin treatments, 1-week-old plants were sprayed with 0.5 μM picloram (synthetic auxin) (Calderón Villalobos et al., 2012; Chapman et al., 2012). For GA treatments, 1-week-old plants were sprayed with 0.01, 0.1, 1 or 100 μM GA3. For leaf-shape analysis, plants were sprayed three times a week, before the initiation of the leaf that was used for phenotyping. For gene expression analysis, plants were sprayed and samples were collected after the indicated times. Specific treatment timings are indicated in the figure legends. Auxin and GA were supplied by Sigma-Aldrich. For paclobutrazol, we used a common agricultural material (Livne et al., 2015; Illouz-Eliaz et al., 2019).

### CRISPR/Cas9 construct design

Constructs were designed to generate deletions within the coding sequence of each target gene, using two sgRNAs alongside the Cas9 endonuclease gene. sgRNAs were designed using the CRISPRp server (http://cbi.hzau.edu.cn/crispr). The constructs for *slga2ox8*, *slga2ox11* and *pslga2ox4* were assembled as detailed by Israeli et al. (2019). For *slga2ox2,4,5*, the constructs were assembled according to Xie et al. (2015). Briefly, the sgRNAs were divided into two parts. Each part was amplified using a sgRNA spacer primer and terminal-specific primers containing a FokI site. After FokI digestion, the fragment was inserted into the BsaI-digested modified pUC57 cloning vector containing a U6 promoter, and subsequently subcloned into the binary vector pMR286. We designed two sgRNAs targeting common sequences in *slga2ox2,4,*5 and recovered several positive T0 plants with the Cas9 cassette. From these lines, we obtained several alleles in the T1 generation. For *SlGA2ox2*, we obtained four different alleles: (1) a 5 bp deletion 354 bp after the ATG, causing a stop codon after 122 amino acids (aa); (2) a 1 bp deletion 356 bp after the ATG, causing a stop codon after 130 aa; (3) a 2 bp deletion 356 bp after the ATG, causing a stop codon after 123 aa; (4) a 155 bp deletion between the two guides, causing a stop codon after 122 aa. For *SlGA2ox4*, we obtained five alleles: (1) a 5 bp deletion 395 bp after the ATG, causing a stop codon after 137 aa; (2) a 1 bp deletion 398 bp after the ATG, causing a stop codon after 137 aa; (3) a 2 bp deletion 398 bp after the ATG causing a stop codon after 138 aa; (4) a 4 bp deletion 396 bp after the ATG causing a stop codon after 136 aa. The fifth allele was obtained from a different construct, targeted to the promoter/upstream region of the *SlGA2ox4* gene. This allele contained a 388 bp deletion, starting from 344 bp upstream to the ATG and ending 44 bp downstream to the ATG, causing a frame shift. For *SlGA2ox5*, we obtained two alleles: (1) a 5 bp deletion 398 bp after the ATG, causing a stop codon after 138 aa; (2) a 5 bp deletion 397 bp after the ATG, causing a stop codon after 138 aa. For *SlGA2ox8*, we obtained one allele with a 118 bp deletion, between the two sgRNAs, 37 bp after the ATG, causing a stop codon after 152 aa. Owing to the close linkage between *SlGA2ox4* and *SlGA2ox5*, the single *Slga2ox4* mutant was a different allele than that used for the double and triple mutants. For *SlGA2ox11*, we obtained two alleles: (1) a 104 bp deletion between the two guides, 253 bp after the ATG, causing a stop codon after 84 aa; (2) a 1 bp deletion (T), 252 bp after the ATG and a 51 bp deletion, 317 bp after the ATG, causing a stop codon after 124 aa. For a description of the different *slga2ox* mutant alleles, see also Fig. S3.

### Imaging

Images of young leaf primordia were captured using an Olympus SZX7 stereo microscope, equipped with a Nikon DXM1200 camera and ACTA software, or a Nikon SMZ1270 stereo microscope equipped with a Nikon DS-Ri2 camera and NIS-ELEMENTS software.

### RNA extraction and qRT-PCR analysis

RNA was extracted using the Plant/Fungi Total RNA Purification Kit (Norgen Biotek) according to the manufacturer's instructions, including DNase treatment. cDNA synthesis was performed using the Verso cDNA Kit (Thermo Fisher Scientific) or SuperScript II reverse transcriptase (18064014, Invitrogen) using 1 μg of RNA. qRT-PCR analysis was carried out using a Corbett Rotor-Gene 6000 real-time PCR machine, with SYBR Premix for all other genes. Levels of mRNA were calculated relative to the *EXPRESSED* (*EXP*) or *TUBULIN* (*TUB*) genes as internal controls as follows: in each biological repeat, the expression levels of the assayed gene and *EXP* or *TUB* were separately calculated relative to a standard curve obtained by a dilution series of a reference sample. The gene expression level in each biological repeat was calculated by dividing the gene expression value by that of *EXP/TUB*. Average expression values were then calculated and presented as ‘relative gene expression’. *EXP* has been shown to be expressed at a similar level throughout tomato development (Expósito-Rodríguez et al., 2008). Similar results were obtained when *TUBULIN* (*TUB*) was used as a reference (Shani et al., 2010). Each biological repeat included between three and15 leaf primordia, depending on the developmental stage**.**

### RNAseq analysis of wild-type versus *entire* (*e*) shoot apices

For RNAseq comparing gene expression between wild type and *e* (Fig. 2, Table S2), shoot apices containing the SAM and the four youngest leaf primordia were collected from M82 and *e* plants, in which L4 (the fourth leaf produced by the plant) was at the P4 stage. RNA was extracted and two biological replicates were used for each genotype. Sequencing libraries were prepared according to the Illumina TruSeq RNA protocol and sequenced on an Illumina HiSeq2000 platform. Reads were aligned to the Tomato reference genome sequence SL4.0 (https://solgenomics.net/ftp/tomato_genome/annotation/ITAG4.0_release/) using TopHat v2.0.6 (Kim et al., 2013). In each library, more than 80% of the reads were uniquely aligned to the reference genome. Reads were attributed to genes by ITAG4.0 (https://solgenomics.net/ftp/tomato_genome/annotation/ITAG4.0_release/) using HTseq v0.62 (Anders et al., 2015). Sample normalization and differential expression were performed using DEseq2 (Love et al., 2014). Genes with FDR<0.05 and an absolute LFC of at least 1 were considered to be differentially expressed.

### RNAseq analysis of wild type versus *slmp* in response to auxin treatment

For RNAseq comparing gene expression between wild type and *slmp* in response to auxin (Fig. 4, Table S1), wild-type and *slmp* plants were sprayed with either water (mock) or 1 μM of the auxin analog picloram. One hour after treatment, 10-15 young leaf primordia (leaf number 5 at the P5 stage) were collected. RNA was extracted and sequencing libraries were prepared using Lexigen 3′ Quant-Seq kit, according to the manufacturer's instructions and sequenced using the NextSeq 500 system with 75 bp single read reads. Reads were cleaned from adapters and quality-trimmed with fastp (v0.23.4; Chen et al., 2018) and matched to the *S. lycopersicum* transcriptome (ITAG4.0) with salmon (v1.10.3; Patro et al., 2017). Variation in gene expression was analyzed with a general linear model in R with the package DESeq (v1.38.3; Love et al., 2014) according to a 2×2 factorial design (Schmid, 2017). Thus, there were three comparisons: (1) auxin treatment in the wild type, (2) auxin treatment in the mutant, and (3) the interaction. The latter tests whether genes respond differently to the auxin treatment according to the genetic background. Within each comparison, *P*-values were adjusted for multiple testing (Benjamini–Hochberg), and genes with an adjusted *P*-value (false discovery rate, FDR) below 0.05 and a minimal log2 fold-change (i.e. the difference between the log2-transformed, normalized sequence counts) of 1 were considered to be differentially expressed. Normalized sequence counts were calculated accordingly with DESeq2 and log2(*x*+1) transformed. To functionally characterize gene sets, we tested for enrichment of gene ontology (GO) terms using topGO (v2.50; Alexa et al., 2006) in conjunction with the GO annotation generated with PANNZER2 (Törönen et al., 2018). Analysis was based on gene counts using the ‘weight’ algorithm with Fisher's exact test (both implemented in topGO). Only terms with at least five genes were used (minimal node size=5). A term was identified as significant if the *P*-value was below 0.05.

### Chlorophyll measurements

Relative chlorophyll levels were quantified using a SPAD chlorophyll meter and displayed in arbitrary SPAD units. Each biological repeat was the mean of three measurements of the third leaf of young seedlings.

### Leaf phenotyping and quantification

Phenotyping and quantification of leaf form was performed on field- or greenhouse-grown plants. Representative mature intact, fifth leaves were photographed using a Nikon D5200 camera. The photographs were used for quantification of leaf-shape phenotype. Leaflet order was defined and leaflet number counted as described before (Shani et al., 2010; Yanai et al., 2011; Bar et al., 2015). Briefly, primary leaflets are separated by a rachis, and some of them develop secondary and tertiary leaflets. Intercalary leaflets are lateral leaflets that develop from the rachis later than the primary leaflets and between them. Each genotype was represented by at least three biological replicates, consisting of leaves from different plants. Mean values were statistically analyzed using the Student's *t*-test. LAS AF and ImageJ software were used for analysis, quantification and measurements of captured images. Student's *t*-test (two-tailed) was used for comparison of means, which were deemed significantly different at *P*≤0.05. Images were manipulated uniformly using Adobe Photoshop. Spacing index was calculated by measuring leaf length and dividing it by the number of primary and intercalary leaflets.

## Supplementary Material



10.1242/develop.205772_sup1 Supplementary information

Table S1. Transcriptomic analysis of *slmp* and wild-type leaf primordia in response to auxin. **S1A: Differential gene expression**. RNA-sequencing data comparing global gene expression profiles between P5 leaf primordia of wild-type (WT) and *slmp* mutants under control (mock) conditions and after 1 hour of auxin treatment. Each genotype-treatment combination is represented by three independent biological replicates shown in separate columns. Values represent normalized sequence counts calculated using DESeq2 and log2(x+1) transformed.
**S1B: Gene Ontology (GO) enrichment analysis**. Functional analysis and GO terms significantly enriched for the interaction effect between genotype (WT vs. *slmp*) and treatment (mock vs. auxin). Only terms represented by at least 5 genes are listed (minimal node size = 5). A term was identified as significant if the P-value was below 0.05.

Table S2. Transcriptomic analysis of *entire (e)* and wild-type shoot apices. **S2A: Differential gene expression**. RNA-sequencing data comparing global gene expression profiles between wild-type (WT) and *entire (e)* mutant shoot apices containing the shoot apical meristem (SAM) and the four youngest leaf primordia (m+p4). Each genotype is represented by two independent biological replicates shown in separate columns. Values represent normalized sequence counts calculated using DESeq2.
**S2B: Gene Ontology (GO) enrichment analysis**. Functional analysis and GO terms significantly enriched based on 414 highly up-regulated genes in the *entire* mutant compared to wild type. Only terms represented by at least 5 genes were used (minimal node size = 5). A term was identified as significant if the P-value was below 0.05.

Table S3. Quantitative RT-PCR analysis of *SlGA2ox* gene expression in response to auxin treatment. **Table S3A: Induction of *SlGA2ox4* expression by short auxin treatment**. qRT-PCR analysis of elative *SlGA2ox4* mRNA level in wild-type shoot apices (m+P5) following treatment with different concentrations of auxin (0.1, 0.5, or 1 μM) and at different time points after treatment (10 minutes or 1 hour), relative to the *EXP* reference gene.
**Table S3B: Induction of *SlGA2ox5* expression by auxin treatment**. qRT-PCR analysis of relative *SlGA2ox5* mRNA level in wild-type shoot apices (m+P5) following 1 hour of auxin treatment, relative to the *EXP* reference gene.
**Table S3C:** Induction of *SlGA2ox4* expression by auxin treatment. qRT-PCR analysis of relative of *SlGA2ox4* mRNA level in wild-type shoot apices (m+P5) following 1 hour of auxin treatment, relative to the *EXP* reference gene.
**Table S3D: Induction of *SlGA2ox3* expression by auxin treatment**. qRT-PCR analysis of relative *SlGA2ox3* mRNA level in wild-type shoot apices (m+P5) following 1 hour of auxin treatment, relative to the *EXP* reference gene.

Table S4. Primers used in this study. List of the primers used for cloning, CRISPR and qRT-PCR.
