# Supplementary material for: A two-step auxin–GA cross-talk regulates organ formation in tomato
Source: Development. 2026 Jul 30;153(14):dev205772. doi: 10.1242/dev.205772 (PMC13489554; doi:10.1242/dev.205772)
Supplement: Supplementary file 1 [file develop-153-205772-s1.pdf]

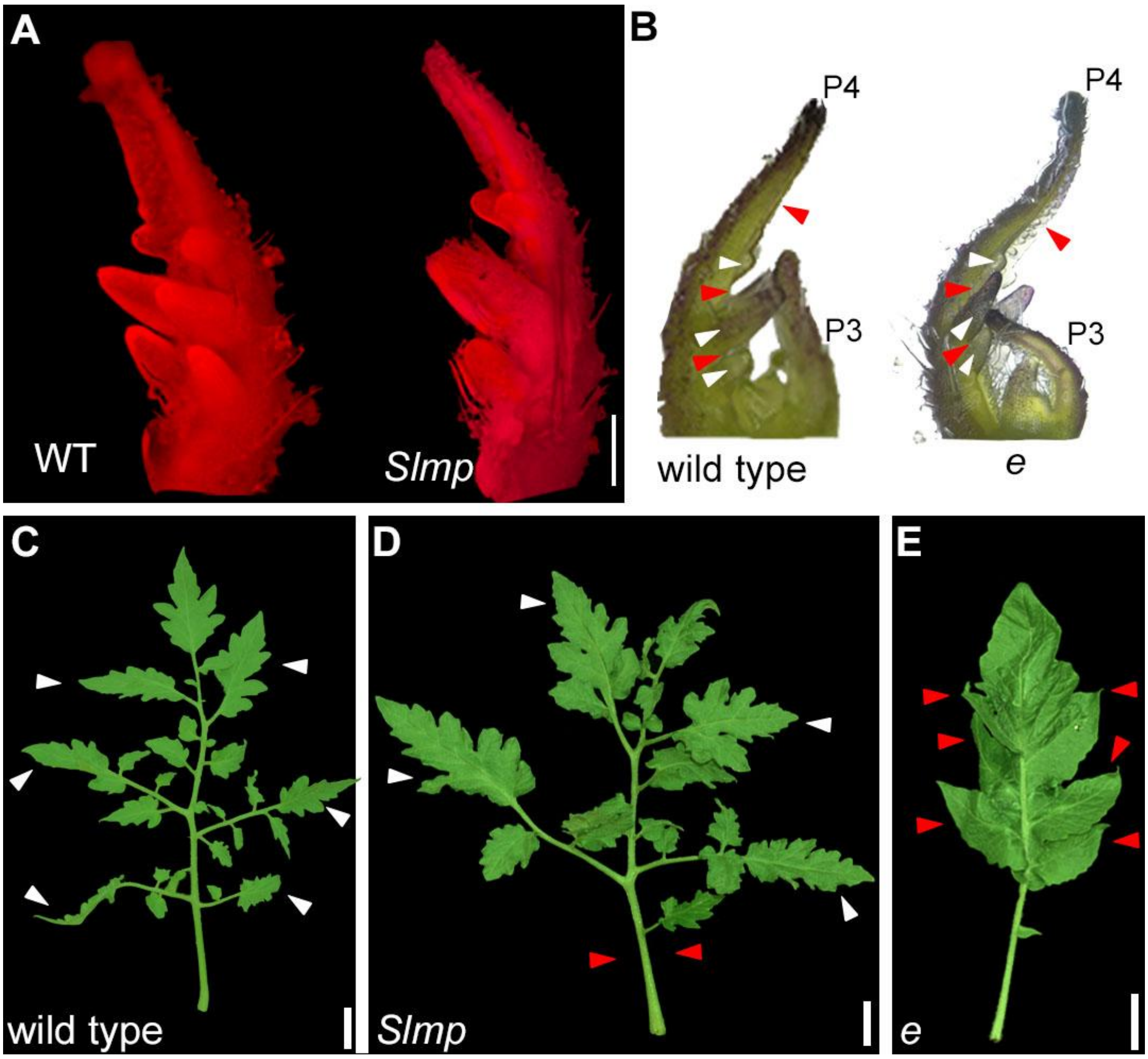

Fig. S1. Images of early leaf primordia and the adult leaves used for the transcriptome proffiling.

(A) Stereomicroscope images showing representative young leaf primordia of the indicated genotypes at the P5 stage, as used for the RNAseq experiment. Scale bar: 200 µm.

(B) Stereomicroscope images showing the shoot apical meristem (SAM) and young leaf primordia (m+P4) of wild type and *e* plants, as used for the RNAseq analysis. White and red arrowheads represent primary leaflets and the intercalary domain, respectively.

(C-E) Mature 5th leaves of the indicated genotypes. White arrowheads point to primary leaflets. Red arrowheads point to missing leaflets or fused leaflets in the intercalary domain. Scale bars: 2 cm.

Development • Supplementary information

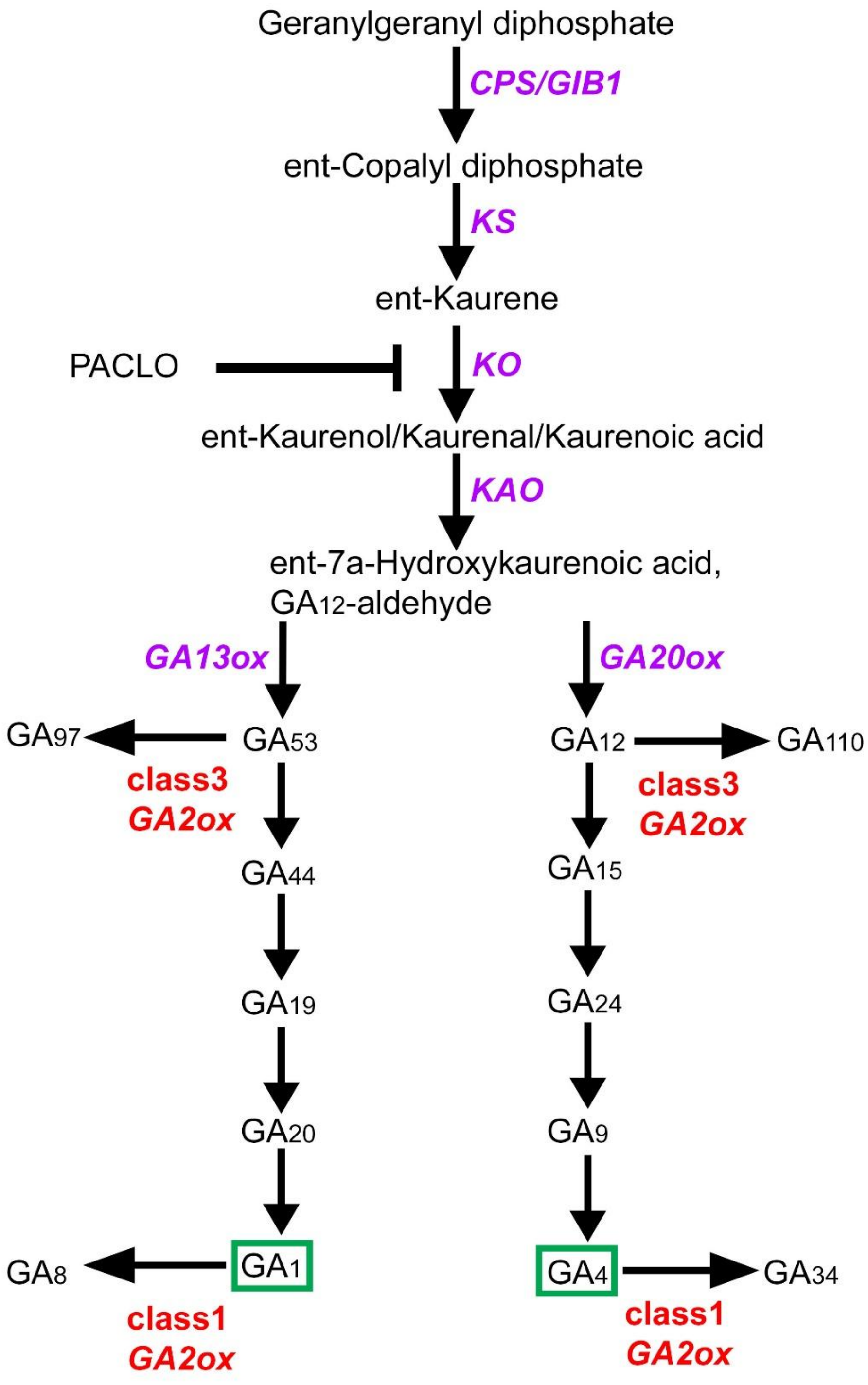

Fig. S2. The GA biosynthesis pathway.

Schematic depiction of the gibberellin biosynthetic pathway. GA intermediates are in black, genes encoding GA-biosynthetic and degradation enzymes are in purple and red, respectively. The two active GAs: GA1 and GA4, are framed by green rectangles.

Development • Supplementary information

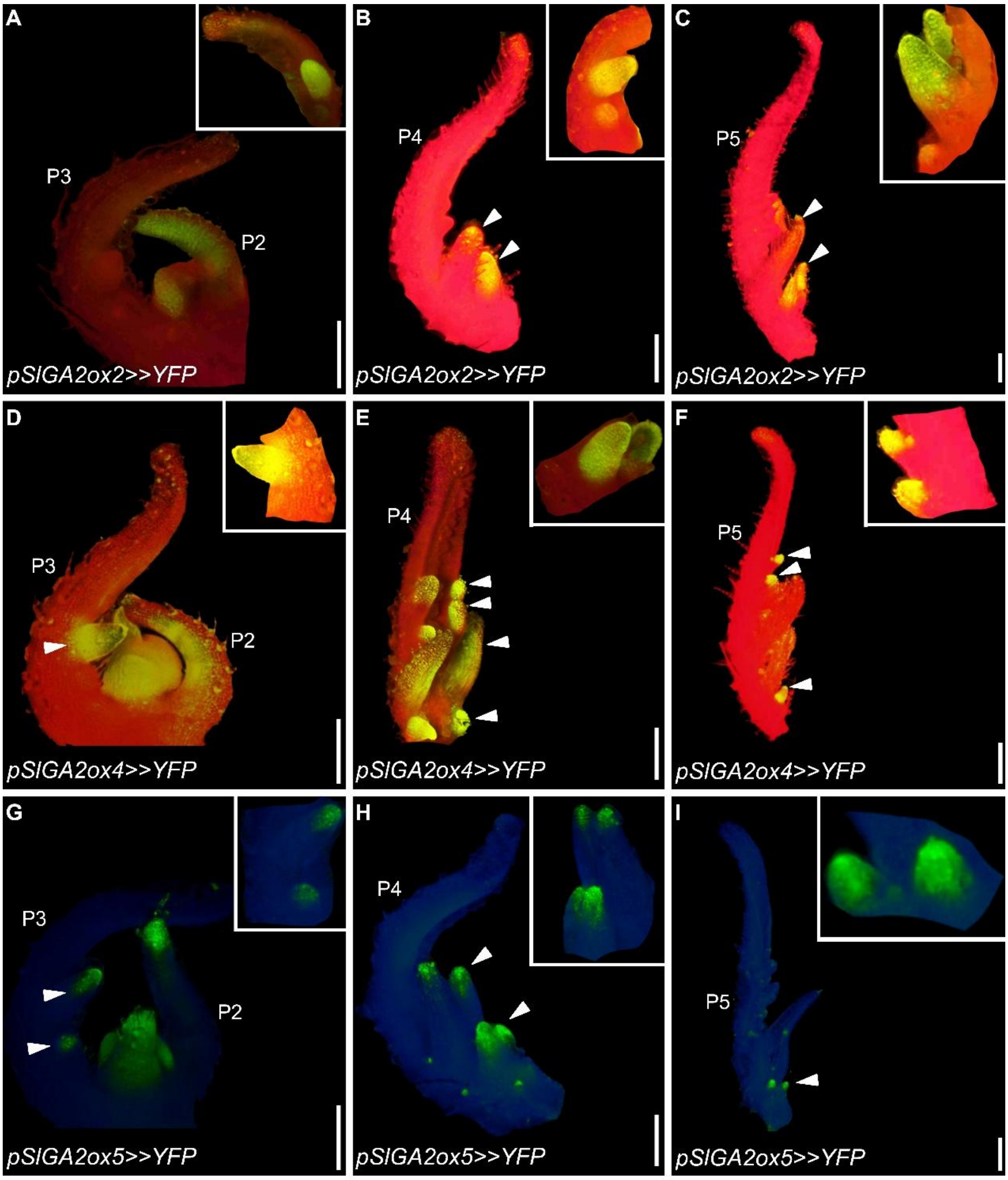

Fig. S3. *SlGA2ox* promoters are expressed at leaflet initiation sites.

(A-I) Stereomicroscope images showing *pSlGA2ox2>>YFP* (A-C), *pSlGA2ox4>>YFP* (D-F) and *pSlGA2ox5>>YFP* (G-I) expression in wild-type young shoot apices and leaf primordia. White arrowheads point to initiating leaflets. Insets show close ups of leaflet initiation sites of the relevant stage. Scale bars: 200 µm.

Development • Supplementary information

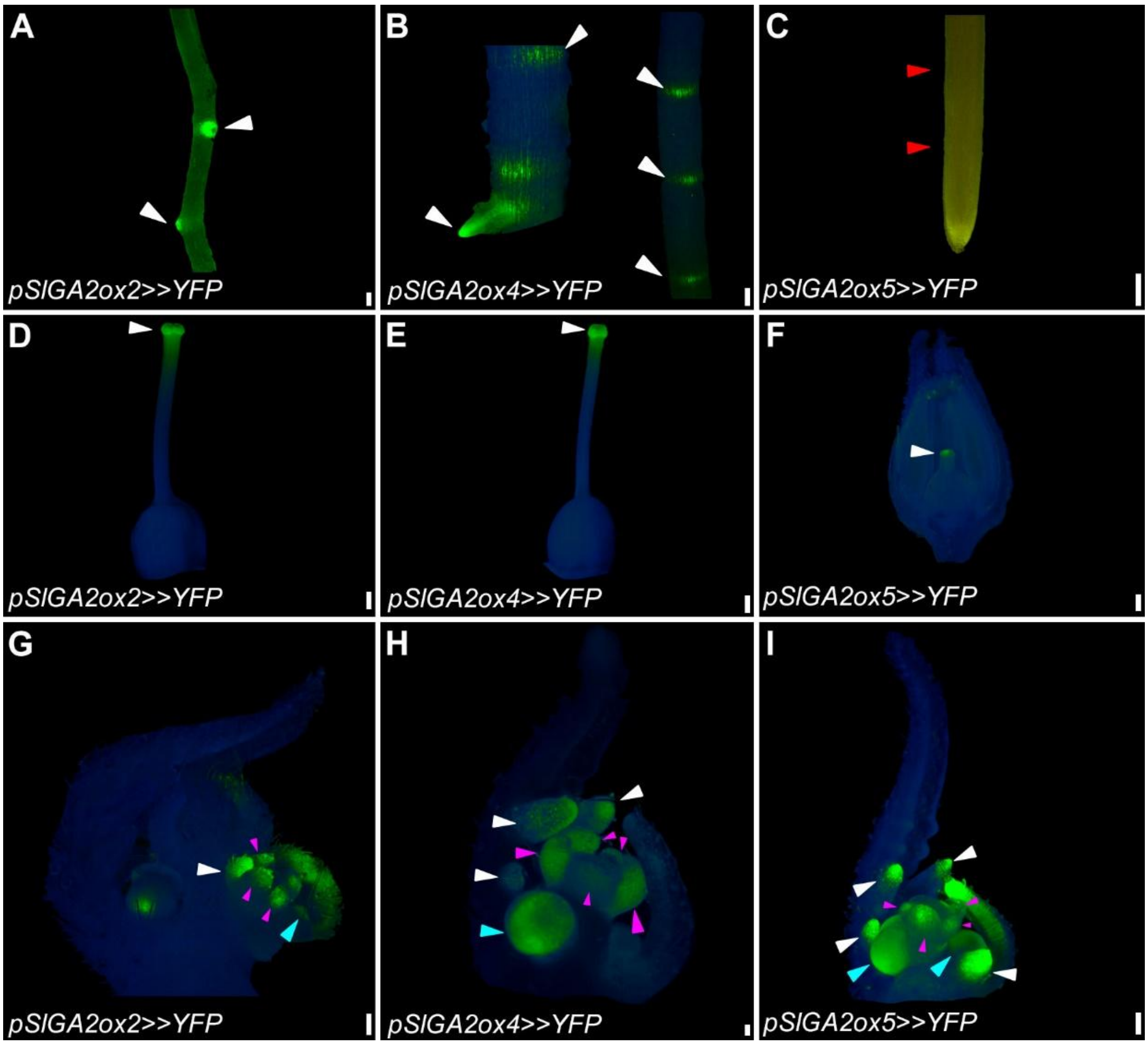

Fig. S4. *pSlGA2ox2,4,5* expression marks the initiation of different organs.

(A-I) Stereomicroscope images showing (A, D, G) *pSlGA2ox2>>YFP* (B, E, H) *pSlGA2ox4>>YFP* and (C, F, I) *pSlGA2ox5>>YFP* expression in the initiation of lateral roots (A-C), flower stigma (D-F) and initiating sepal and young reproductive meristems (G-I). Azure arrowheads point to the different reproductive meristems (transition meristem, flower meristem and sympodial meristem). White arrowheads point to initiating leaves, leaflets and lateral roots. Pink arrowheads point to initiating sepals. Red arrowheads point to missing expression of the *pSlGA2ox5* in lateral roots. Scale bars: A and I – 1mm. B – 10 µm. C – 0.5 mm. D, E, F, G and H – 100 µm.

Development • Supplementary information

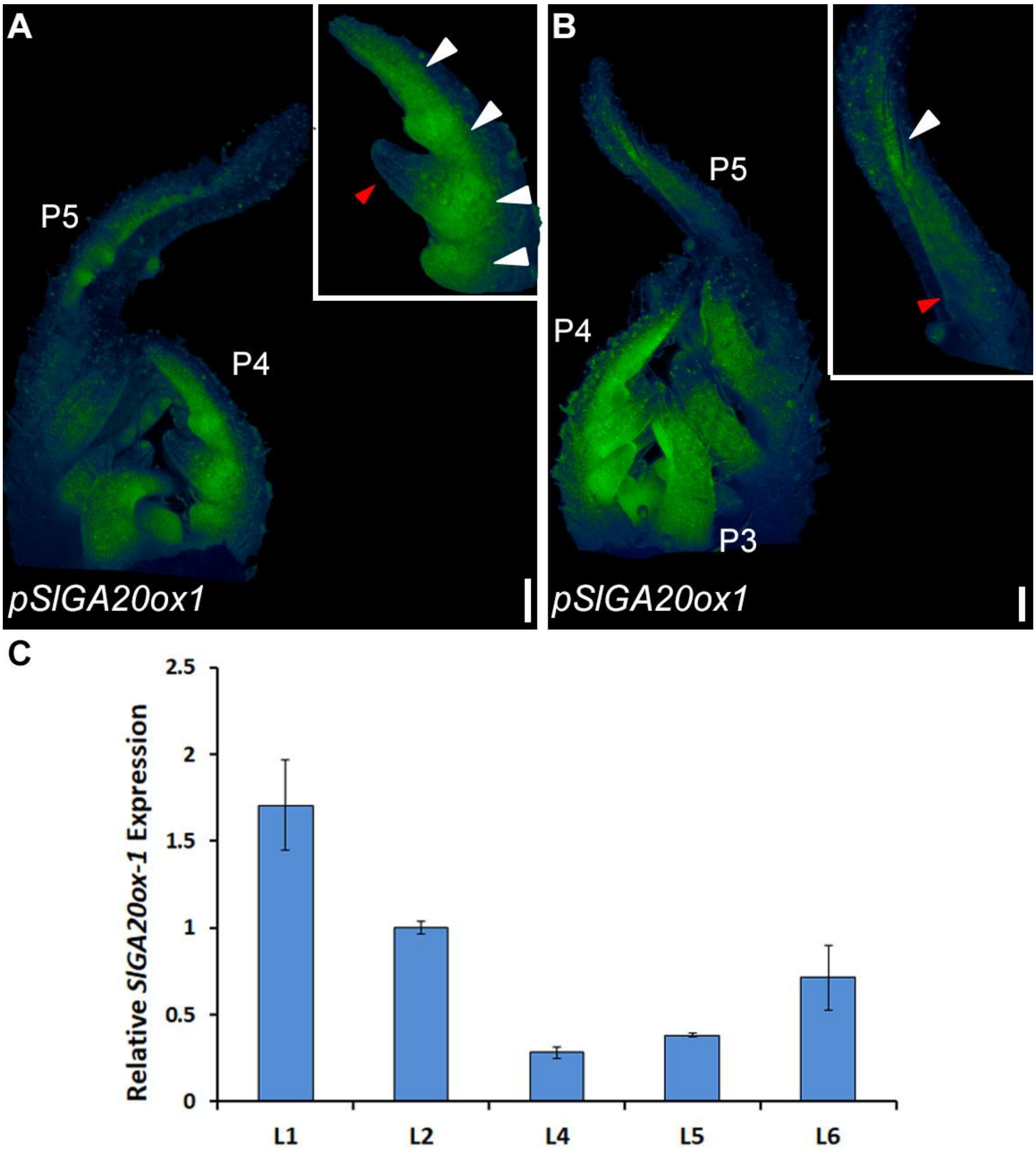

Fig. S5. p*SlGA20ox1* is expressed in growing areas.

(A-B) Stereomicroscope images showing *pSlGA20ox1>>RFP* expression in the wild-type SAM and young leaf primordia. White arrowheads point to growing areas. Red arrowheads point to regions of reduced *pSlGA20ox1* expression. Scale bars: 200 µm.

(C) qRT-PCR analysis of *SlGA20ox1* mRNA expression in wild-type P5 leaf primordia of the indicated leaves, relative to the *EXP* reference gene. Bars represent the SE of at least three biological replicates, each containing between 1–3 plants.

Development • Supplementary information

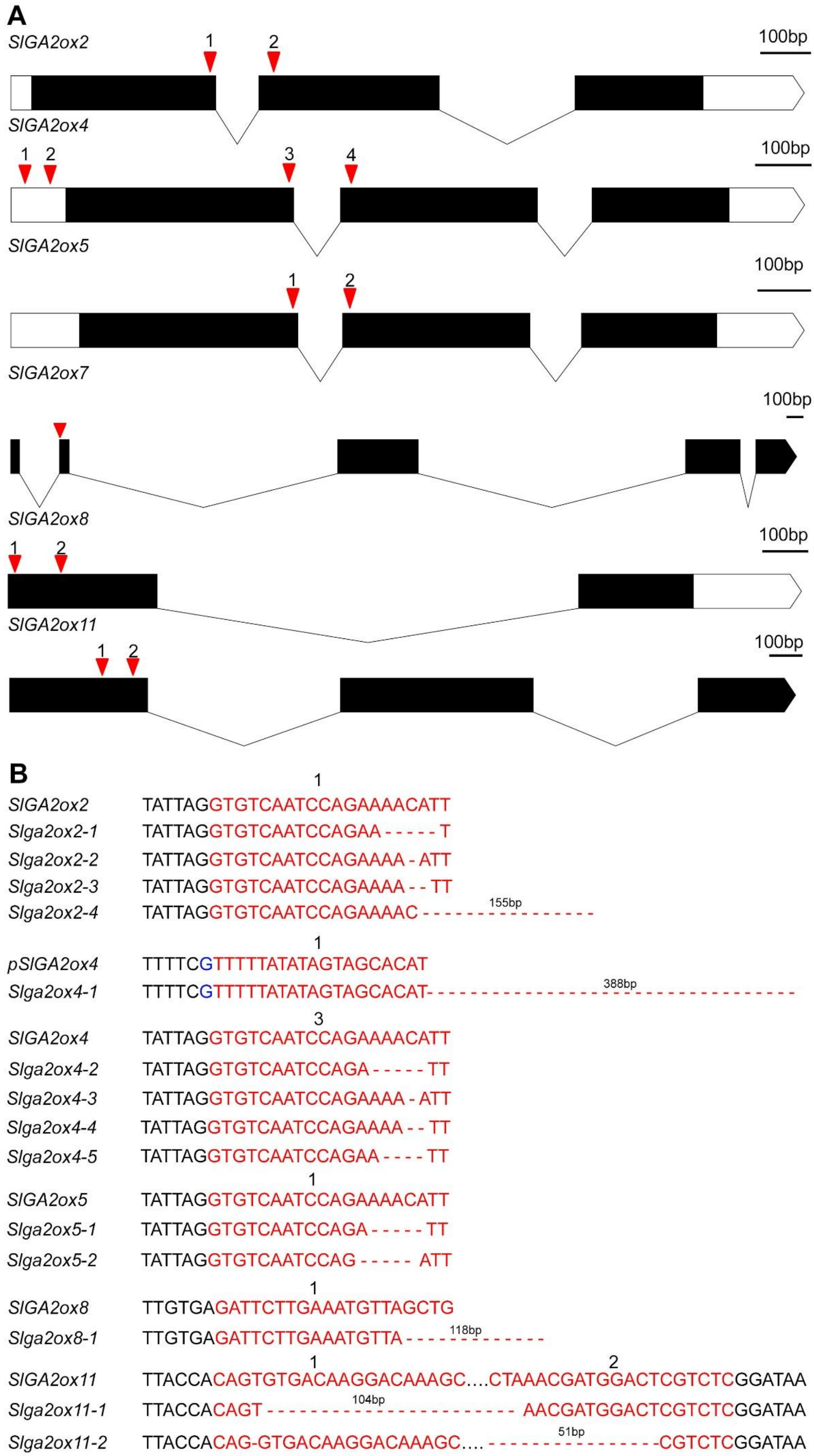

Development • Supplementary information

Fig. S6. The *slga2ox* alleles generated and used in the study.

(A) Schemes of the *SlGA2ox2, SlGA2ox4, SlGA2ox5, SlGA2ox7 SlGA2ox8* and *SlGA2ox11* genes. Arrowheads indicate the locations of the guide RNAs used for CRISPR to generate the different alleles.

(B) Sequences of the different *SlGA2ox2*, *SlGA2ox4*, *SlGA2ox5*, *SlGA2ox8* and *SlGA2ox11* CRISPR alleles. The wild-type sequence of the relevant region is shown on the top row, and the sgRNA sequences are in red. Blue letters indicate mismatches between the gDNA and sgRNA.

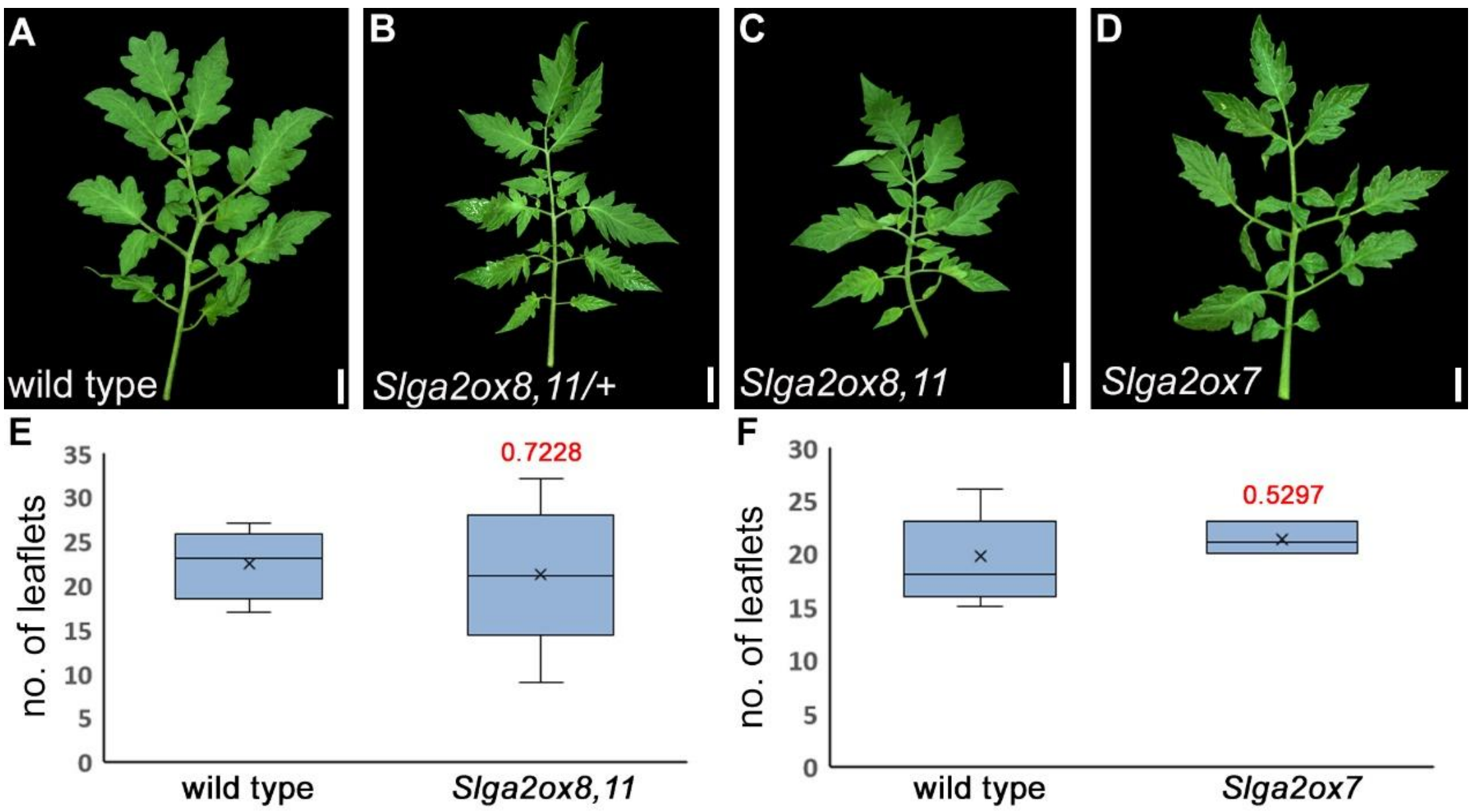

Fig. S7. Additional *slga2ox* mutants.

Mutations in *slga2ox7, 8* and *11* have normal leaflet number.

(A-D) Mature 5th leaves of the indicated genotypes. Scale bars: 2 cm.

(E, F) Quantification of the total number of leaflets in the indicated genotypes. Error bars represent SE of at least 5 leaves. Statistical significance was assessed using unpaired two-tailed Student’s t-tests.

Development • Supplementary information

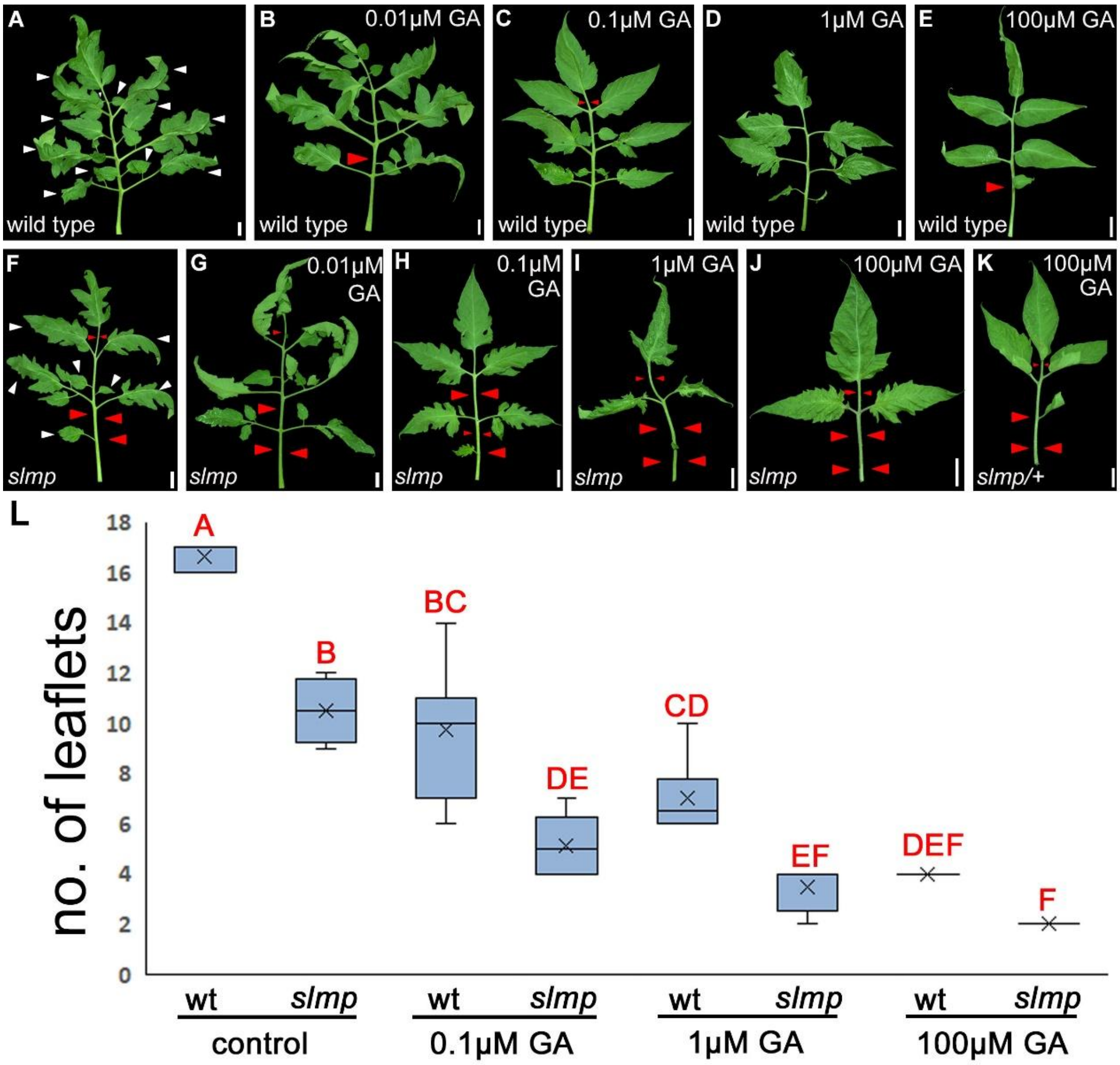

Fig. S8. GA application phenocopies the reduced leaflet phenotype of *slmp*.

(A-K) Mature 5th leaves of the indicated genotypes and treatments. White arrowheads point to primary or intercalary leaflets, and red arrowheads to missing leaflets. GA3 was applied to plants by spraying. GA concentrations are indicated in μM. in Scale bars: 2 cm.

(L) Quantification of the total number of leaflets in the indicated genotypes in (A-K). Error bars represent SE of at least 5 leaves; Different letters indicate statistically significant differences determined by one-way ANOVA followed by Tukey–Kramer's multiple comparisons test ($P < 0.05$).

Development • Supplementary information

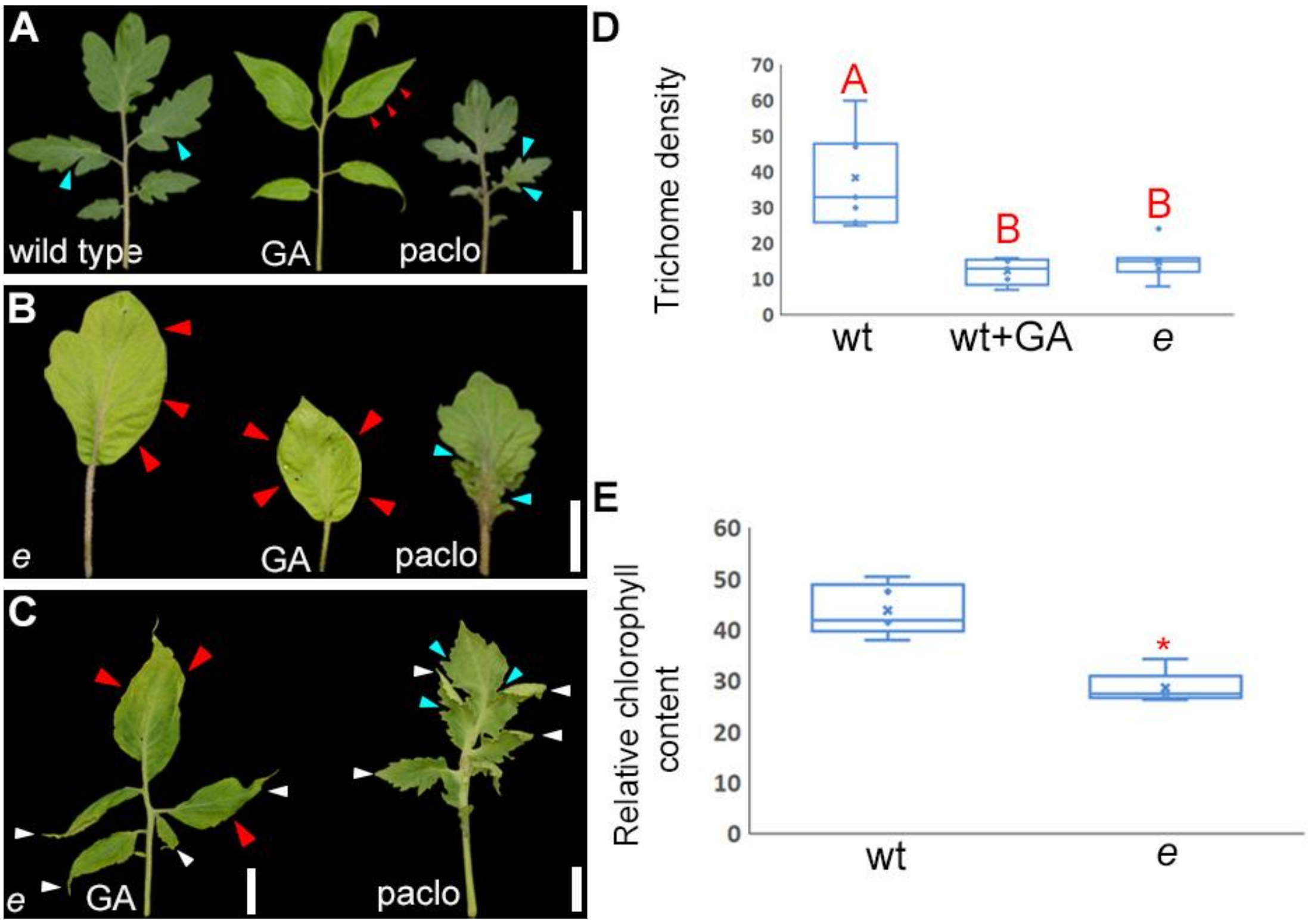

Fig. S9. Common phenotypes casued by enahnced auxin and GA response in tomato leaves.

(A-C) Young (A-B) and mature (C) 3rd leaves of the indicated genotypes and treatments. Azure, red and white arrowheads point to lobe sinuses, smooth margins and separated primary leaflets, respectively. In older leaves, both GA and paclobutrazol suppress the *e* simple leaf phenotype. Scale bars: 2 cm. Statistical significance was assessed using unpaired two-tailed Student's t-tests.

(D) Trichome density was determined by counting trichomes within a 4 × 4 mm square on the third mature leaf. For each genotype and/or treatment, at least three squares per leaf and at least three leaves were analyzed, and values represent the mean. Different letters indicate statistically significant differences determined by one-way ANOVA followed by Tukey–Kramer's multiple comparisons test ($P < 0.05$).

(E) Relative chlorophyll content was measured using a SPAD chlorophyll meter (arbitrary SPAD units); each biological replicate is the mean of at least three measurements from the third leaf of young seedlings. Statistical significance was assessed using unpaired two-tailed Student's t-tests.

Development • Supplementary information

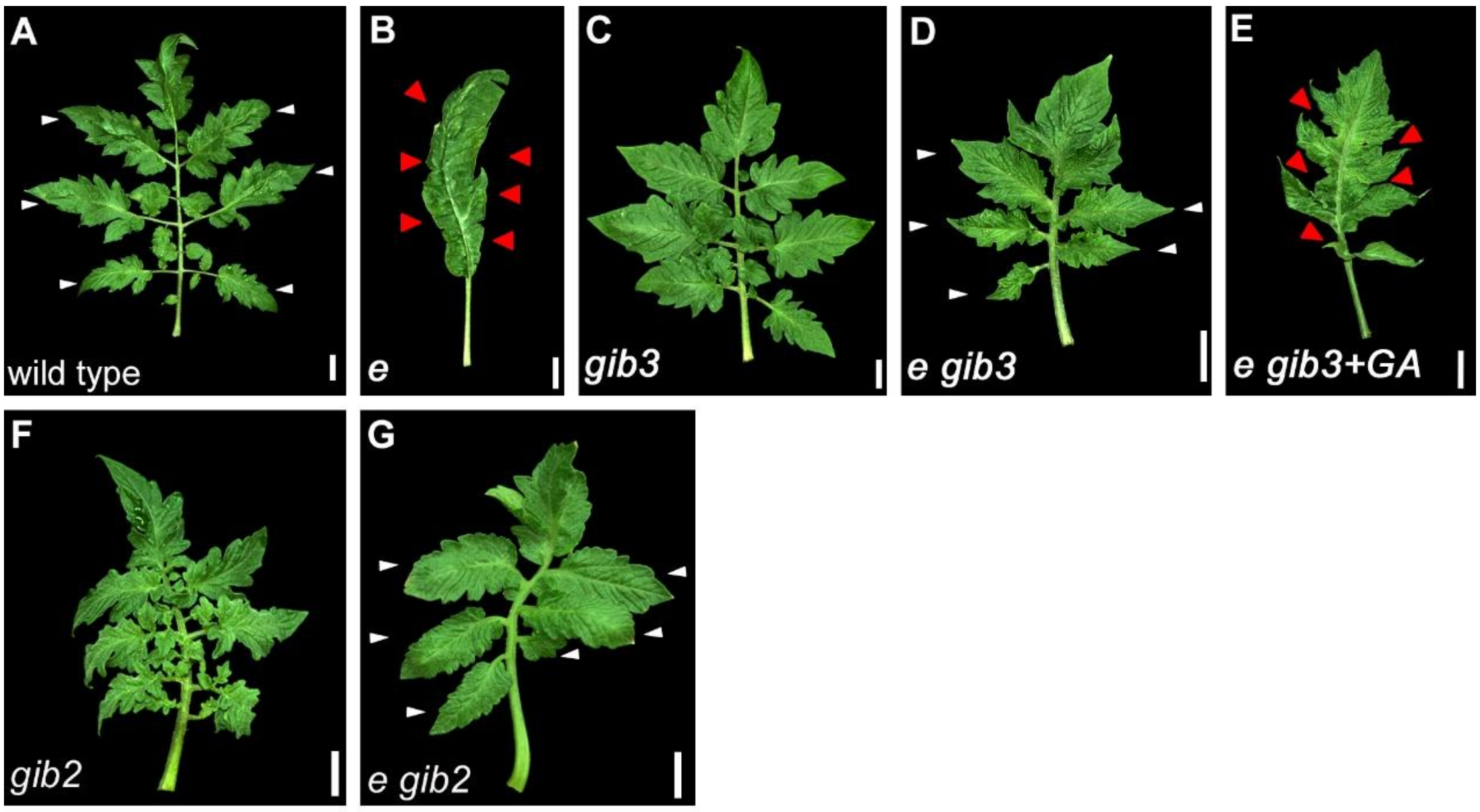

Fig. S10. *e* ectopic blade growth is mediated by GA.

(A-G) Mature 5th leaves of the indicated genotypes. Scale bars: 2 cm. White arrowheads point to primary or intercalary leaflets and red arrowheads to fused ectopic blade in the intercalary domain. The GA-deficient mutants *gib2* and *gib3* suppress the ectopic blade growth of *e*, with the double mutant *e gib2* and *e gib3* producing separated primary leaflets. GA application restore the ectopic blade growth of *e gib3* mutant.

Development • Supplementary information

Table S1. Transcriptomic analysis of *slmp* and wild-type leaf primordia in response to auxin.

S1A: Differential gene expression. RNA-sequencing data comparing global gene expression profiles between P5 leaf primordia of wild-type (WT) and *slmp* mutants under control (mock) conditions and after 1 hour of auxin treatment. Each genotype-treatment combination is represented by three independent biological replicates shown in separate columns. Values represent normalized sequence counts calculated using DESeq2 and log2(x+1) transformed.

S1B: Gene Ontology (GO) enrichment analysis. Functional analysis and GO terms significantly enriched for the interaction effect between genotype (WT vs. *slmp*) and treatment (mock vs. auxin). Only terms represented by at least 5 genes are listed (minimal node size = 5). A term was identified as significant if the P-value was below 0.05.

Available for download at
https://journals.biologists.com/dev/article-lookup/doi/10.1242/dev.205772#supplementary-data

Table S2. Transcriptomic analysis of *entire* (*e*) and wild-type shoot apices.

S2A: Differential gene expression. RNA-sequencing data comparing global gene expression profiles between wild-type (WT) and *entire* (*e*) mutant shoot apices containing the shoot apical meristem (SAM) and the four youngest leaf primordia (m+p4). Each genotype is represented by two independent biological replicates shown in separate columns. Values represent normalized sequence counts calculated using DESeq2.

S2B: Gene Ontology (GO) enrichment analysis. Functional analysis and GO terms significantly enriched based on 414 highly up-regulated genes in the *entire* mutant compared to wild type. Only terms represented by at least 5 genes were used (minimal node size = 5). A term was identified as significant if the P-value was below 0.05.

Available for download at
https://journals.biologists.com/dev/article-lookup/doi/10.1242/dev.205772#supplementary-data

Development • Supplementary information

Table S3. Quantitative RT-PCR analysis of *SlGA2ox* gene expression in response to auxin treatment.

Table S3A: Induction of *SlGA2ox4* expression by short auxin treatment. qRT-PCR analysis of elative *SlGA2ox4* mRNA level in wild-type shoot apices (m+P5) following treatment with different concentrations of auxin (0.1, 0.5, or 1 μM) and at different time points after treatment (10 minutes or 1 hour), relative to the *EXP* reference gene.

Table S3B: Induction of *SlGA2ox5* expression by auxin treatment. qRT-PCR analysis of relative *SlGA2ox5* mRNA level in wild-type shoot apices (m+P5) following 1 hour of auxin treatment, relative to the *EXP* reference gene.

Table S3C: Induction of *SlGA2ox4* expression by auxin treatment. qRT-PCR analysis of relative of *SlGA2ox4* mRNA level in wild-type shoot apices (m+P5) following 1 hour of auxin treatment, relative to the *EXP* reference gene.

Table S3D: Induction of *SlGA2ox3* expression by auxin treatment. qRT-PCR analysis of relative *SlGA2ox3* mRNA level in wild-type shoot apices (m+P5) following 1 hour of auxin treatment, relative to the *EXP* reference gene.

Available for download at
https://journals.biologists.com/dev/article-lookup/doi/10.1242/dev.205772#supplementary-data

Table S4. Primers used in this study. List of the primers used for cloning, CRISPR and qRT-PCR.

Available for download at
https://journals.biologists.com/dev/article-lookup/doi/10.1242/dev.205772#supplementary-data

Development • Supplementary information
