# Supplementary material for: A two-step auxin–GA cross-talk regulates organ formation in tomato
Source: Development. 2026 Jul 30;153(14):dev205772. doi: 10.1242/dev.205772 (PMC13489554; doi:10.1242/dev.205772)
Supplement: Supplementary file 2 [file develop-153-205772_review_history.pdf]

A two-step auxin—GA cross-talk regulates organ formation in tomato

Alon Israeli, Dov Nir, Ido Shwartz, Matan Levy, Marc W. Schmid, Yogev Burko, Idan Efroni and Naomi Ori

DOI: 10.1242/dev.205772

Editor: Dominique Bergmann

Review timeline

Original submission:	30 April 2026
Editorial decision:	23 May 2026
First revision received:	17 June 2026
Accepted:	18 June 2026

Original submission

First decision letter

MS ID#: dev.205772

MS TITLE: A two-step auxin-GA cross talk regulates organ formation

AUTHORS: Naomi Ori; Alon Israeli; Dov Nir; Ido Shwartz; Matan Levy; Marc W. Schmid; Yogev Burko; Idan Efroni

Dear Dr Ori,

I have now received all the referees reports on the above manuscript, and have reached a decision. The referees' comments are appended below.

The overall evaluation is positive and we would like to publish a revised manuscript in Development, provided that the referees' comments can be satisfactorily addressed. Please attend to all of the reviewers' comments in your revised manuscript and detail them in your point-by-point response. If you do not agree with any of their criticisms or suggestions explain clearly why this is so. If it would be helpful, you are welcome to contact us to discuss your revision in greater detail. Please send us a point-by-point response indicating your plans for addressing the referees' comments, and we will look over this and provide further guidance.

Please upload both a 'clean' version of your Word file, along with a highlighted version clearly showing

Reviewer 1

This paper is a collaboration of excellent, accomplished scientists, who know how to design experiments, understand the salient data points and write well. It was a fun read. The conclusions are logically rendered and the figures are mostly excellent.

The concept that auxin signaling can render different effects on GA biology at different developmental stages of leaf ontogeny is well supported, and explained in the informed context of tomato leaf and leaflet development. The summary model in Figure 6 is simple and elegant I have just a few suggestions.

© 2026. Published by The Company of Biologists under the terms of the Creative Commons Attribution License (https://creativecommons.org/licenses/by/4.0/).

1. Figure 2. Are there companion images of later stages in leaf primordium development that show high expression of GA biosynthetic markers - in keeping with the proposed model?

2. Figure 3. The Illustration in E is extrapolative. How are the "GAOX activities" (displayed in the gradient scale) measured, quantified as shades of green in the mutants and the wild type plants? The phenotype is significant but is not dramatic.

3. Why not show the actual leaves instead of artistic renderings in Fig1 and. Fig 3, when showing mutant phenotypes versus wild type? This has become fashionable but why not show the plants?

Reviewer 2

SUMMARY OF THE ADVANCE MADE IN THIS PAPER AND ITS POTENTIAL SIGNIFICANCE TO THE FIELD

The authors investigate the interplay of gibberellin and auxin in tomato compound leaf development. The key idea advanced, is that there is a dual, context-dependent interaction between these two hormones in organ development: auxin reduces GA to enable leaflet initiation, and later induces GA to allow leaflet growth. Overall, the manuscript presents very interesting observations based on generating novel genetic material including CRISPR mutants in GA catabolism genes and transgenic lines where domain-specific reduction of GA activity is shown to cause increased leaflet number. The questions addressed are important both in terms of developmental control of organ shape, and interactions between plant hormones and their developmental significance. The manuscript is overall of high quality and very interesting to read, and the conclusions are overall supported by the data. In some instances, the reasoning isn't fully clear (at least to me) and in my opinion some aspects of the argumentation would benefit from rethinking and being put in the context of prior work in the field. The comments below are meant to help clarify these points and to make the narrative/argumentation tighter.

SUGGESTIONS TO AUTHORS

The authors start with transcriptome analysis in response to exogenous auxin and in mutants with perturbed auxin signalling. A key observation is that GA catabolism genes are induced following short-term auxin treatment while in the entire mutant, which shows elevated/broadened auxin signalling, the opposite effect is seen, as GA biosynthetic gene expression is elevated and GA catabolic gene expression is downregulated, indicating that a long-term increased auxin response leads to increased GA activity. The authors conclude that auxin affects GA metabolism genes during leaflet development in two phases, the first predicted to lead to reduced GA activity at leaflet initiation, and the second predicted to lead to increased GA activity during blade growth. I can see the point that short term local application has different effects to longer term elevated auxin response, but don't fully see the tie-in of the spatial aspect here i.e. the initiation vs blade outgrowth. The reasoning could be explained more here I think, and while this conclusion seems reasonable if one also considers later experiments (eg the domain specific perturbations), it may be premature at this stage of the narrative. Similarly, Fig3 is titled "GA2ox are involved in auxin and SlMP-mediated leaflet formation". I can see the effect on leaflet number and it is a very nice result, but what is the tie-in to SlMP/auxin here? Finally, in lines 198-205 the authors state "If reduced GA is important for leaflet initiation downstream of auxin and SlMP, then GA application might lead to a reduced number of leaflets". The issue here is that it is already well known that GA application or elevated GA signalling reduces leaflet number-why not simply discuss the prior work in relation to their gene expression rather than presenting this GA application as a new experiment. Of course, the authors here can discuss that they verified earlier data which is good to see, and discuss the GA/Slmp experiment. On this last point (GA/Slmp) a sentence more of interpretation might be useful here -how can we be sure that the results reflect a linear pathway as the authors seem to indicate?

Th entire gib double mutant phenotype is very nice and suggests that ectopic blade growth in response to increased auxin activity depends on GA which is important for the authors conclusions. Perhaps this can be discussed a bit more.

© 2026. Published by The Company of Biologists under the terms of the Creative Commons Attribution License (https://creativecommons.org/licenses/by/4.0/).

The authors then use a two-component transactivation system to show that reduced GA activity resulting from expressing of a truncated DELLA or GA2Ox4 is sufficient to increase leaflet number. This is a really great result. One aspect that I think would be good to discuss here is to put this information in the context of previous work (Jasinski et al The Plant Journal 2005) showing that gib mutants do not show increased leaflet number but that they produced more secondary leaflets per primary leaflet than wild-type, and strongly enhance of leaflet number phenotypes of KNOX gain of function mutants. What could these differences between gib mutants and targeted perturbation of GA activity mean? That the genetic manipulations here condition stronger reductions in local GA levels or signalling? Does the juxtaposition of "low" and "high" GA regions here (but not in gib) cause a stronger effect on leaflet number? Is it simply environmental variation (seems unlikely but did the authors consider?). The Jasinski TPJ paper also discusses effects of GA activity on cell growth, and the consequences for leaf shape and the competence to form leaflets which seem relevant for the discussion.

For the two component results it would be good to mention how many transgenic lines were scored for the responder construct. Apologies if I missed it.

The imaging is OK as is but would benefit by showing closer up views of the developmentally relevant areas at least for the most critical points- if it is practical for the authors to do that.

There is a typo in line 365 (Cardamine hirsuta) and grammar for the last sentence (line 366 onwards) needs another look.

Reviewer 3

The plant hormone auxin plays an important role on organ initiation and differential growth stages. However, it is unclear what is the underlying molecular pathways integrating the auxin signaling into these two continuous stages of organ formation. In this manuscript, the author Israeli et al., show that auxin promotes leaflet initiation and growth via opposite effects on gibberelin (GAï¼‰ metabolism. The short auxin treatment experiment show the GA catabolism and biosynthesis genes are affected significantly, while a steady state increase in auxin response or long auxin treatment induced the expression of GA biosynthesis genes. Genetic data support that reduction of GA biosynthesis suppressed auxin-induced blade growth. Leaflet number was reduced by the increased GA activity, and local reduction of GA activity led to the formation of more leaflets. It suggests the leaflet initiation requires reduced GA. Transgenic plants with a GA catabolism gene in initiating leaflets was sufficient to induce ectopic leaflets in the adjacent, intercalary region, suggesting a non-autonomous function of GA in leaflet initiation. Taken together, this study reveals a context-dependent dual interaction between auxin and GA in leaflet formation: auxin reduced GA to enable initiation, and to allow growth. It will be very helpful to understand the interactions between plant organ initiation and plant hormones.

I have some *Comments for the author*:

1. These is a small mistake in the figure 2 Legend part: In the figure 2 (A-C) Stereomicroscope/confocal images showing pSlGA2ox2, pSlGA2ox4 and pSlGA2ox5 driving an YFP reporter, in wild-type SAM and young leaf primordia. Based on the figures, it should be in young leaf primordia, not included wild type SAM.

2. In the Figure S6, GA3 was applied to tomato plants by spraying. Please add the concentration on the 0.1 GA, 1 GA and 100 GA , respectively.

First revision

Author response to reviewers' comments

Dear Dr. Bergmann,

© 2026. Published by The Company of Biologists under the terms of the Creative Commons Attribution License (https://creativecommons.org/licenses/by/4.0/).

We thank you and the reviewers for the careful evaluation of our manuscript, and are grateful for the positive and constructive comments.

We have carefully revised the manuscript in response to all comments. Specifically, in the revised version we:

Clarified the temporal and spatial interpretation of auxin-GA interactions.

Expanded the discussion in relation to previous studies.

Added additional developmental images and figure clarifications.

Revised figure legends.

Corrected specific errors, omissions and unclarities.

Updated the manuscript to conform to Development formatting guidelines. Below is a detailed point-by-point response to each reviewer comment.

Reviewer 1

We thank Reviewer 1 for the positive assessment of our work and for the constructive suggestions.

Figure 2. Are there companion images of later stages in leaf primordium development that show high expression of GA biosynthetic markers - in keeping with the proposed model?

We have added two new supplementary figures, S3 and S5, showing additional developmental stages for *pSlGA2ox2*, *pSlGA2ox4*, *pSlGA2ox5*, and *pSlGA20ox1* during leaflet initiation and early blade growth. For the three *SlGA2ox* reporters, the images clearly demonstrate high local expression at initiating leaflets from stages P3 through P5. For *pSlGA20ox1*, we added a later stage (P5) showing robust expression in growing zones, though slightly reduced compared to the expansion phase at P4.

To further address the timing issue, we also used qRT-PCR to evaluate *SlGA20ox1* expression across successive leaves at the P5 stage (new Figure S5C), representing the developmental windows of primary leaflet initiation and blade growth, which is the focus of our model (P3–P5). Due to heteroblastic development, early tomato leaves, such as leaf 1 and 2 (the first and second leaves formed by the plant) progress through their morphogenetic and growth windows much more rapidly than later leaves (Leaves 4–6). Consistent with our hypothesis that *SlGA20ox1* is involved in tissue expansion, Leaf 1 exhibits significantly higher expression at P5 compared to the slower-developing later leaves at the identical developmental stage. We hope that these additional images and experiments further support our working hypothesis and clarify the timing issue.

Figure 3. The Illustration in E is extrapolative. How are the "GAOX activities" (displayed in the gradient scale) measured, quantified as shades of green in the mutants and the wild type plants? The phenotype is significant but is not dramatic.

We agree that the figure was conceptual and is not based on quantitative data of GA2ox activity. Therefore, in the revised manuscript we removed the hypothetical schematic illustration (formerly Figure 3E).

Why not show the actual leaves instead of artistic renderings in Fig1 and. Fig 3, when showing mutant phenotypes versus wild type? This has become fashionable but why not show the plants?

We agree that in Figure 3 the use of illustrations was not essential. Therefore, in the revised version we removed the schematic renderings from Figure 3E and kept the images of the actual leaves. In addition, representative leaves for the analyzed genotypes in the transcriptomic experiment have been added to Figure S1C-E. Representative images of the actual leaves of the

© 2026. Published by The Company of Biologists under the terms of the Creative Commons Attribution License (https://creativecommons.org/licenses/by/4.0/).

analyzed genotypes are presented throughout the manuscript in the context of the different experiments.

In Figure 1 however, our goal was to emphasize the distinction between the intercalary and growth domains, as well as the differences between leaflet initiation and blade growth in the WT, *slmp*, and *e* genotypes. We therefore chose in this case to keep the simplified black silhouettes of actual leaves to help the reader focus specifically on these differences rather than on additional phenotypes.

Reviewer 2

We thank Reviewer 2 for the encouragement, the thorough reading and the thoughtful and detailed comments, which helped us improve the clarity and framing of the manuscript.

The authors start with transcriptome analysis in response to exogenous auxin and in mutants with perturbed auxin signaling. A key observation is that GA catabolism genes are induced following short-term auxin treatment while in the entire mutant, which shows elevated/broadened auxin signaling, the opposite effect is seen, as GA biosynthetic gene expression is elevated and GA catabolic gene expression is downregulated, indicating that a long-term increased auxin response leads to increased GA activity. The authors conclude that auxin affects GA metabolism genes during leaflet development in two phases, the first predicted to lead to reduced GA activity at leaflet initiation, and the second predicted to lead to increased GA activity during blade growth. I can see the point that short term local application has different effects to longer term elevated auxin response, but don't fully see the tie-in of the spatial aspect here i.e. the initiation vs blade outgrowth. The reasoning could be explained more here I think, and while this conclusion seems reasonable if one also considers later experiments (eg the domain specific perturbations), it may be premature at this stage of the narrative.

We appreciate this important point and agree that the logic connecting the transcriptomic observations with the developmental interpretation required clarification. In the revised manuscript, we now explain more explicitly, already at the beginning of the Results section, the developmental basis for our interpretation. Specifically, we clarify that during normal tomato leaf development, leaflet initiation precedes blade growth, and that this developmental progression forms the basis for separating the initiation and growth phases in our model. In addition, we have added clarifying sentences highlighting why we think that the distinct transcriptomic profiles of short-term auxin treatment versus the steady-state *entire (e)* mutant, reflect these sequential developmental phases. Finally, we have added a concluding summary paragraph at the end of this section to explicitly connect the temporal transcriptomic data with our spatiotemporal model of leaflet initiation and blade expansion. We hope this clarifies the conceptual framework.

Similarly, Fig3 is titled "GA2ox are involved in auxin and SlMP-mediated leaflet formation". I can see the effect on leaflet number and it is a very nice result, but what is the tie-in to SlMP/auxin here?

We agree with this comment and revised the title to '*Mutations in SlGA2ox genes reduce leaflet number*'.

Finally, in lines 198-205 the authors state "If reduced GA is important for leaflet initiation downstream of auxin and SlMP, then GA application might lead to a reduced number of leaflets". The issue here is that it is already well known that GA application or elevated GA signaling reduces leaflet number-why not simply discuss the prior work in relation to their gene expression rather than presenting this GA application as a new experiment. Of course, the authors here can discuss that they verified earlier data which is good to see, and discuss the GA/Slmp experiment.

We agree that it is known that GA activity reduces leaflet number and that the results in this section should have been placed in the context of previous literature regarding the effect of GA on leaflet number. We apologize for this. In the revised manuscript, we now first cite and discuss earlier studies showing that GA application or elevated GA signaling during early leaf development leads to reduced leaflet number, likely by accelerating maturation and shortening the

© 2026. Published by The Company of Biologists under the terms of the Creative Commons Attribution License (https://creativecommons.org/licenses/by/4.0/).

morphogenetic window during which leaflet initiation occur. In the revised text, we clarify that our experiments intended to test whether GA may additionally function within the morphogenetic window to regulate leaflet initiation locally.

On this last point (GA/Slmp) a sentence more of interpretation might be useful here -how can we be sure that the results reflect a linear pathway as the authors seem to indicate?

We agree that the results do not distinguish between a linear and a more complex pathway. We added a sentence clarifying this.

The *entire gib* double mutant phenotype is very nice and suggests that ectopic blade growth in response to increased auxin activity depends on GA which is important for the authors conclusions. Perhaps this can be discussed a bit more.

To emphasize the importance of the *entire gib* double mutant phenotype and its significance, we have expanded our Discussion section and explicitly integrated the underlying rationale into the Results section.

The authors then use a two-component transactivation system to show that reduced GA activity resulting from expressing of a truncated DELLA or GA2Ox4 is sufficient to increase leaflet number. This is a really great result. One aspect that I think would be good to discuss here is to put this information in the context of previous work (Jasinski et al The Plant Journal 2005) showing that gib mutants do not show increased leaflet number but that they produced more secondary leaflets per primary leaflet than wild- type, and strongly enhance of leaflet number phenotypes of KNOX gain of function mutants. What could these differences between gib mutants and targeted perturbation of GA activity mean? That the genetic manipulations here condition stronger reductions in local GA levels or signaling? Does the juxtaposition of "low" and "high" GA regions here (but not in gib) cause a stronger effect on leaflet number? Is it simply environmental variation (seems unlikely but did the authors consider?). The Jasinski TPJ paper also discusses effects of GA activity on cell growth, and the consequences for leaf shape and the competence to form leaflets which seem relevant for the discussion.

In the revised manuscript, we now discuss this earlier work showing that *gib1* mutants do not increase total leaflet number but enhance KNOX gain-of-function phenotypes (Jasinski et al., 2005). Global reduction of GA biosynthesis affects overall growth, leaf elongation, and maturation, reflecting the well-established role of GA in cell expansion and differentiation. These broad developmental effects may partially mask the more local role of GA in regulating leaflet initiation within the morphogenetic window. We suggest that the local genetic manipulations used in the present study enabled the separation of two distinct roles of GA during leaf development. We now discuss these possibilities in the revised Discussion section.

For the two component results it would be good to mention how many transgenic lines were scored for the responder construct. Apologies if I missed it.

In the revised manuscript, we added to the information in the Materials and Methods section the transgenic lines used and their origin in more detail:

For the *op:PROΔ17* responder line, we used a previously published line described in (Nir *et al.*, 2017). Two independent lines were characterized with different promoters and showed similar phenotypes with varying severity. To avoid variability resulting from differences in transgene strength, one representative line was selected and used consistently throughout all crosses and experiments.

For the *pE:LhG4* driver line, we used a previously published line described in (Israeli *et al.*, 2019). Two independent lines were tested in a cross with *op:PROΔ17* and showed similar phenotypes with varying severity. To avoid variability resulting from differences in transgene strength, one representative line was selected and used consistently throughout all crosses and experiments.

For the *op:GA2ox4* responder line, we used a previously published line described in (*Yanai et al., 2011*), which was used consistently throughout the study.

© 2026. Published by The Company of Biologists under the terms of the Creative Commons Attribution License (https://creativecommons.org/licenses/by/4.0/).

For the *pDR5:LhG4* driver line, we used a line showing expression patterns similar to the direct fluorescent *DR5:VENUS* reporter described previously (Shani *et al.*, 2010; Ben-Gera *et al.*, 2012).

The imaging is OK as is but would benefit by showing closer up views of the developmentally relevant areas at least for the most critical points- if it is practical for the authors to do that.

In the revised manuscript, we have included close-ups of the relevant regions in the *pSlGA2ox2, 4,* 5 images of the primary leaflet initiation regions (new Figure S3) and in the *pSlGA20ox1* images of the blade growth regions (new Figure S5).

There is a typo in line 365 (Cardamine hirsuta) and grammar for the last sentence (line 366 onwards) needs another look.

We corrected the typo in *Cardamine hirsuta* and revised the final sentence.

Reviewer 3

We thank Reviewer 3 for the encouraging feedback and helpful suggestions.

There is a small mistake in the figure 2 legend.

We corrected the Figure 2 legend to indicate that panels A-C show young leaf primordia and not the SAM.

In Figure S6 Please add the concentration of the GA that was applied.

We thank the reviewer for noticing this omission. We added the GA3 concentrations to Figure S6 (now S8) and clarified the treatment conditions in the Materials and Methods section.

Additional changes:

We shortened the abstract according to the instructions.

We added a supplementary table listing the primers used in the study.

We slightly altered Figure S6 to more clearly describe the mutant alleles.

Sincerely

Alon Israeli and Naomi Ori, on behalf of all authors.

References

Ben-Gera H, Shwartz I, Shao M-R, Shani E, Estelle M, Ori N. 2012. ENTIRE and GOBLET promote leaflet development in tomato by modulating auxin response. *The Plant journal* 70: 903-915.

Israeli A, Capua Y, Shwartz I, Tal L, Meir Z, Levy M, Bar M, Efroni I and, Ori **N. 2019**. Multiple Auxin-Response Regulators Enable Stability and Variability in Leaf Development. *Current Biology* **29**: 1746–1759.

Jasinski S, Tattersall A, Piazza P, Hay A, Martinez-Garcia JF, Schmitz G, Theres K, McCormick S, Tsiantis M. 2008. PROCERA encodes a DELLA protein that mediates control of dissected leaf form in tomato. *The Plant journal : for cell and molecular biology* **56**: 603-12.

Nir I, Shohat H, Panizel I, Olszewski NE, Aharoni A, Weiss D. 2017. The Tomato DELLA Protein PROCERA Acts in Guard Cells to Promote Stomatal Closure. *The Plant Cell* **29**: 3186–3197.

© 2026. Published by The Company of Biologists under the terms of the Creative Commons Attribution License (https://creativecommons.org/licenses/by/4.0/).

Shani E, Ben-Gera H, Shleizer-Burko S, Burko Y, Weiss D, Ori N. 2010. Cytokinin regulates compound leaf development in tomato. *The Plant cell* **22**: 3206– 3217.

Yanai O, Shani E, Russ D, Ori N. 2011. Gibberellin partly mediates LANCEOLATE activity in tomato. *The Plant Journal* **68**: 571–582.

<u>Second decision letter</u>

MS ID#: dev.205772R1

MS TITLE: A two-step auxin-GA cross talk regulates organ formation

AUTHORS: Naomi Ori; Alon Israeli; Dov Nir; Ido Shwartz; Matan Levy; Marc W. Schmid; Yogev Burko; Idan Efroni

Dear Dr Ori,

I am happy to tell you that your manuscript has been accepted for publication in Development, pending our standard publication integrity checks.

© 2026. Published by The Company of Biologists under the terms of the Creative Commons Attribution License (https://creativecommons.org/licenses/by/4.0/).
